# MICAL, the Flavoenzyme Participating in Cytoskeleton Dynamics

**DOI:** 10.3390/ijms14046920

**Published:** 2013-03-27

**Authors:** Maria A. Vanoni, Teresa Vitali, Daniela Zucchini

**Affiliations:** Department of Biosciences, University of Milan, Via Celoria 26, Milano 20133, Italy; E-Mails: teresa.vitali@unimi.it (T.V.); rzucchini@yahoo.it (D.Z.)

**Keywords:** MICAL, flavoprotein, NADPH oxidase, monooxygenase, semaphorin signaling, actin dynamics

## Abstract

MICAL (from the Molecule Interacting with CasL) indicates a family of recently discovered cytosolic, multidomain proteins, which uniquely couple an *N*-terminal FAD-containing monooxygenase-like domain to typical calponine homology, LIM and coiled-coil protein-interaction modules. Genetic and cell biology approaches have demonstrated an essential role of the catalytic activity of the monooxygenase-like domain in transducing the signal initiated by semaphorins interaction with their plexin receptors, which results in local actin cytoskeleton disassembly as part of fundamental processes that include differentiation, migration and cell-cell contacts in neuronal and non-neuronal cell types. This review focuses on the structure-function relations of the MICAL monooxygenase-like domain as they are emerging from the available *in vitro* studies on mouse, human and Drosophila MICAL forms that demonstrated a NADPH-dependent actin depolymerizing activity of MICAL. With Drosophila MICAL forms, actin depolymerization was demonstrated to be associated to conversion of Met44 to methionine sulfone through a postulated hydroxylating reaction. Arguments supporting the concept that MICAL effect on F-actin may be reversible will be discussed.

## 1. Introduction

MICAL (from the Molecule Interacting with CasL) indicates a family of recently discovered multidomain proteins [1,2], which participate in the control of cytoskeleton dynamics with still poorly understood mechanisms. A peculiar feature of MICAL proteins is the presence of an *N*-terminal region structurally related to the bacterial FAD-containing aromatic monooxygenases [1–4] of which *p*-hydroxybenzoate hydroxylase (PHBH) is the prototype ([5,6] and references therein). Thus, MICALs appear to be unique among proteins involved in cytoskeleton dynamics in that they may establish a direct link between cell oxidoreduction metabolism and cytoskeleton rearrangements, which are at the basis of cell proliferation, migration and differentiation, cell-cell contacts and intracellular vesicle trafficking in health and disease [7]. Most of our knowledge on MICAL function derives from genetic and cell biology studies, which have been presented in several recent reviews [8–16], which will be briefly summarized here. Much less is known about the actual structure-function relations of MICALs as derived from *in vitro* biochemical studies of isolated protein forms, which are the focus of this article.

## 2. Discovery and Hypotheses on MICAL Function

### 2.1. Discovery of MICALs

**Figure 1:**
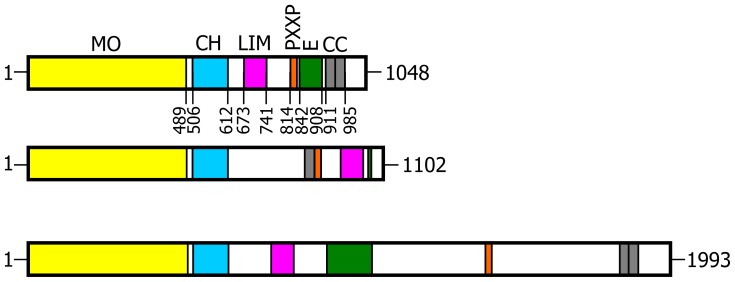
Domain organization of Molecule Interacting with CasL (MICAL) forms. The boundaries of the domains are referred to the mouse forms. From top to bottom: MICAL1, MICAL2 and MICAL3. See text for details.

During the search for CasL SH3 domain interactors among proteins recombinantly expressed from a thymus cDNA library, Suzuki *et al*. [1] identified a novel cytoplasmic protein, which they named MICAL1 from the Molecule Interacting with CasL. CasL (also known as HEF1 and NEDD9) belongs to the Cas family of proteins. It is a docking protein important for several functions among which are T cell receptor and β1 integrin-induced responses such as interleukin-2 production and migratory response, which all involve cytoskeleton rearrangements. Sequence analysis of the cDNA encoding MICAL revealed that it is a multidomain protein coupling an *N*-terminal flavoprotein-like domain to regions known to mediate protein-protein interactions, namely: a calponin homology (CH) domain typical of actin binding proteins; a LIM domain (from the three gene products Lin-11, Isl-1 and Mec-3, [17,18]), which contains two zinc fingers; a Pro-rich region with the PXKP signature for the interaction with SH3 domains; a *C*-terminal region with motifs suggesting the formation of coiled-coil structures (Figure 1). Co-localization and co-immunoprecipitation experiments, following expression of various MICAL1 constructs in the cells, demonstrated that MICAL1 interacts with CasL through its Pro-rich region [1]. MICAL1 was also found to co-localize with vimentin, the main component of intermediate filaments, and to interact with it with its *C*-terminal region [1]. Although the interaction with vimentin still awaits independent confirmation [8,19], these observations led to the proposal that MICAL1 participates in the cascade of events downstream of CasL phosphorylation in response to β1 integrin and/or T cell receptor stimulation, providing a physical link with cytoskeleton proteins [1]. Independent studies in the Kolodkin group on the intracellular components that transduce the signal initiated by the interaction of semaphorins with their plexin receptors on neurons led to identify MICAL1 as a novel interactor of PlexA cytoplasmic domain in an embryonic Drosophila cDNA library [2]. While in Drosophila there is only one gene encoding MICAL, vertebrates contain three genes encoding MICAL isoforms indicated as MICAL1, MICAL2 and MICAL3 (Figure 1) [1,2,20]. Furthermore, MICAL-like forms, also encoded by different genes, have been identified [1,2,21,22], but they will not be discussed further due to the absence of the flavoprotein region. Genetic studies showed that inactivation of the single MICAL gene of Drosophila leads to phenotypes similar to those obtained by inactivating either the semaphorin 3A (Sema3A) or plexin A (PlexA) [2]. By introducing the rather drastic triple G-to-W substitution in the GXGXXG motif in the unique putative adenylate-binding site of MICAL flavoprotein-like *N*-terminal domain, it was also concluded that the integrity of such domain is required for MICAL function [2]. This conclusion was independently confirmed by Beuchle *et al*. [23] who correlated point mutations in the Drosophila MICAL gene, all causing a milchstrasse phenotype, with alterations of myofilaments organization and synaptic structure. Evidence in support of the essentiality of the *N*-terminal region for MICAL function was later obtained also in mammalian neuronal and non-neuronal cells [24,25].

Overall, these studies demonstrated a role of MICAL in transducing the signal initiated by the interaction of semaphorins with their plexin receptors on the target cells, which leads to local (actin) cytoskeleton disassembly, as exemplified by axon growth cone collapse [2]. They also provided the first evidence that the *N*-terminal putative flavoprotein domain may be essential for MICAL function [2,23]. The latter is not restricted to neuronal cells as demonstrated by, e.g., the original work of Suzuki *et al*. in T cells [1], that of Beuchle *et al*. [23] in Drosophila somatic muscle cells, of Fisher *et al*. [19] in rat and by the more recent experiments of Giridharan *et al*. [25] in non-neuronal mammalian cells.

The recent review of Hung *et al*. [9] provides an excellent historical overview on the discovery of semaphorins as chemorepellent protein factors capable to induce the collapse of the axon growth cone through local disassembly of actin filaments and, therefore, to control the direction of axon growth towards its target cell. The review also nicely describes the experimental approaches that led to the identification and characterization of several proteins participating in the transduction of semaphorin signaling. With other recent articles [8,16], it also summarizes the evidence for the role, distribution and expression patterns of MICAL forms as gathered through genetic and cell biology studies on different cell types. The original discovery of chemorepellent and chemoattractive cues stimulated a variety of studies aiming to understand how cytoskeleton dynamics is controlled, at the molecular level, in different cells. Such studies led to the discovery of a large number of proteins, including GTPases, GTPase-activating proteins and GTP-binding proteins, the microtubule regulators tau and the collapsin response mediator proteins (CRMP), several actin polymerizing/depolymerizing and bundling factors and, as a novel member, MICALs. However, in spite of the fact that a great deal of work has already been done, how the dynamics of the cytoskeleton is controlled in the cell is far from being understood. The growing number of interconnected signaling pathways and of the (macro)molecules participating in the process is actually demonstrating the complexity of the control of cytoskeleton dynamics. For example, although the role and mechanism of action of semaphorins has been initially studied in neurons, it is now clear that they participate in the control of the cytoskeleton in a broad variety of cells, through their receptors and downstream effectors. As a result, understanding the molecular details of the semaphorin-initiated signaling pathway is also of interest in order to design ways to modulate their action in the context of neurodegenerative diseases, of cell metastatization and migration, and of angiogenesis as part of cancer therapy, of pathogen infection or to promote axon regeneration in, e.g., spinal cord injury [7–9,11,12,14,26–37]. In this respect, understanding MICAL mechanism of action is important in that it may provide key information for the understanding of the semaphorin pathway as well as tools to control it [11,14,26,31,37]. In support of biomedically relevant outcomes of studies on MICAL are several observations. The expression of MICAL forms increases at sites of spinal cord injury in model animals, thus supporting the hypothesis that inhibiting its function may be beneficial to promote neuronal regeneration [38]. MICAL is also involved in myofilaments organization and synaptic structure [23,25] suggesting that modulation of MICAL activity may be of interest also in the context of neuromuscular disorders, including amyotrophic lateral sclerosis [31]. A link between MICAL function and vesicle trafficking between the endoplasmic reticulum and the Golgi complex was also found [19,39]. Furthermore, the overexpression of MICAL2 alternative splicing variants associates with particularly aggressive prostate cancers forms [37]. Lowering their levels through small interfering RNA technology significantly decreases cell growth [37]. Finally, the work of Xue *et al*. [40] on zebrafish suggests that MICAL forms may also be important for cardiovascular development.

### 2.2. Hypotheses on MICAL Function

**Figure 2:**
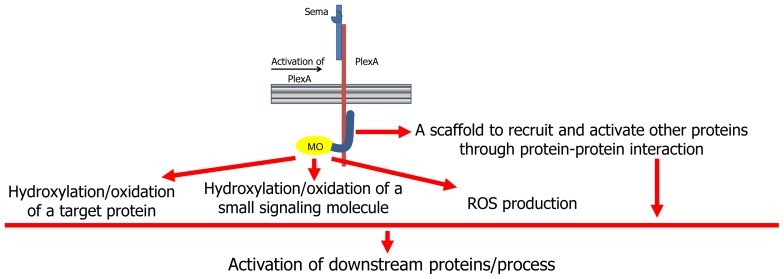
Schematic representation of the proposed functions of MICAL. The scheme is based on [13].

On the basis of the body of current knowledge on MICALs, several hypotheses on its mode of action have been made (Figure 2, [7,9,13]). According to a first hypothesis, MICAL may simply act as a scaffold protein. When the semaphorin (Sema1A in Drosophila, Sema3A in mammals) interacts with its plexin A receptor directly (as in Drosophila) or through the neuropilin coreceptors (in mammals), several proteins are recruited to the (activated) plexin cytoplasmic domain. Among them is MICAL, which interacts with plexin through its *C*-terminal region (Figure 2, [2]).

This scaffold hypothesis is supported by the presence in MICAL of protein interaction domains (Figure 1) and by the observation that MICAL1 binds CasL with its Pro-rich region [1], with the rab 1 GTPase with its last ~145 residues [41] and with CRMP forms with its *N*-terminal region including the flavoprotein, CH and LIM domains [24]. Thus, following semaphorin-plexin interaction, MICAL binds to the cytoplasmic region of plexin where it helps recruiting other proteins and promoting the modulation of their activity with respect to the downstream events (Figure 2).

The scaffold model would explain how MICAL may interfere with CasL [1,8,13] or CRMP [16,24] functions by sequestering them. In support of a scaffold role is the finding that MICAL1 modulates apoptosis by binding NDR1/2 (nuclear Dbf2-related) kinases, through its part spanning from the LIM domain to the *C*-terminus [42]. By competing with MST1 for NDR binding, MICAL prevents mammalian Ste-20-like kinase (MST1)-induced NDR activation [42]. Finally, there are several MICAL-like proteins lacking the *N*-terminal flavoprotein-like region, which also participate in functions linked to cytoskeleton dynamics such as endosomal recycling and establishment of cell-cell contacts [22,43–51].

The presence of the *N*-terminal region with sequence similarity to several flavoproteins of the oxidase and monooxygenase (hydroxylase) class, which appeared to be essential for MICAL function [2], led to alternative hypotheses linking MICAL function to the catalytic activity of its *N*-terminal flavoprotein domain ([13], Figure 2).

This hypothesis is supported by several experiments that showed that the *N*-terminal flavoprotein-like domain of MICAL is essential for its function, while the other domains are important for its localization [2,9,24,25,52,53]. The binding of MICAL to plexin cytosolic region (and/or further interaction with other proteins) would activate MICAL’s enzymatic function.

According to one hypothesis, MICAL flavoprotein domain would catalyze an oxidase reaction by mediating the transfer of reducing equivalents from a small molecule to molecular oxygen with the release of hydrogen peroxide (or superoxide anion) as the signaling molecule responsible of the downstream events [7,9,13,16].

In support of this hypothesis is the finding that CRMP2 may be oxidized in response to semaphorin stimulation leading to formation of a homodimer stabilized by a disulfide bridge [54]. Oxidized CRMP2 may form a transient disulfide-linked complex with thioredoxin, which would stimulate CRMP2 inactivation through phosphorylation by glycogen synthase kinase-3, leading to growth cone collapse [54].

As discussed below, the MICAL flavoprotein-like domain actually exhibits a NAD(P)H oxidase activity, which would be consistent with the hypothesis. As an alternative, the small molecule being oxidized by MICAL would be the actual effector, but no evidence in support of this hypothesis is available. Finally, MICAL could covalently modify, through oxidation or hydroxylation, a protein side chain, leading to a modulation of its function. As discussed below, on the basis of the structure of MICAL’s *N*-terminal region [3,4] and of experimental evidence, actin [52,53] and the collapsin responsive mediator protein-1 (CRMP1) [24] may be good candidates as the protein substrates.

**Figure 3:**
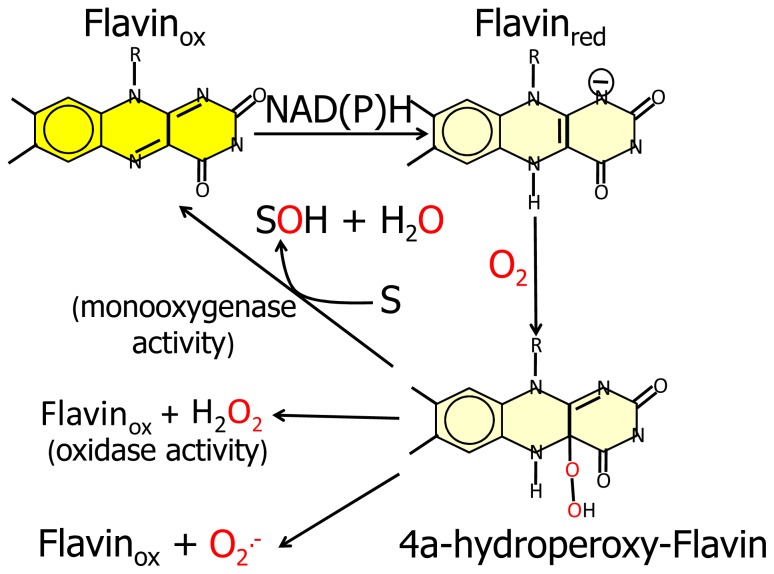
General scheme of the catalytic activities of FAD-dependent monooxygenases (hydroxylases) [6,55].

Furthermore, the similarity of the *N*-terminal protein region with FAD-dependent monooxygenases would suggest that MICAL catalyzes a hydroxylation rather than an oxidase reaction. In this case no reactive oxygen species would be released since it is well known that FAD containing monooxgenases/hydroxylases insert one oxygen atom from molecular oxygen in the hydroxylatable substrate and release the second oxygen atom in a water molecule (Figure 3, [5,6,55]). In this reaction, reducing equivalents would be provided by reduced pyridine nucleotides, NADH or NADPH, possibly linking the control of cytoskeleton dynamics to the primary cell energy metabolism and/or signaling pathways involving NAD derivatives like, e.g., polyADP ribose [56].

Should MICAL modify a protein side-chain, one of the relevant biological issues would be the reversibility of the modification. Cysteine oxidation leading to formation of disulfides may be reversed by systems like that formed by the thioredoxin/thioredoxin reductase couple [57], while methionine oxidation may be reversed by methionine sulfoxide reductases [58]. Modification of other side chains may require novel enzymatic activities, which would lead to other exciting discoveries.

Since FAD-dependent monooxygenases can catalyze a NAD(P)H oxidase reaction in the absence of the substrate to be hydroxylated (Figure 3), it cannot be ruled out that MICALs may differentially control the oxidase and the monooxygenase activities depending on the MICAL isoform and/or the presence of interacting (macro)molecules.

Whether MICAL becomes catalytically active only when it is bound to plexin, or other proteins determine its activation still needs to be established. The work of Schmidt *et al*. [24], later confirmed by Giridharan *et al*. [25], supported the hypothesis that the activity of the *N*-terminal catalytic domain is autoinhibited by the region *C*-terminal to the LIM domain (see below). Binding of plexin A and other interactors to such a *C*-terminal region would remove the inhibition activating MICAL reaction. Furthermore, removal of the physical interaction between the *N*-terminal and the *C*-terminal regions may allow binding of the actual (protein) substrate to the *N*-terminal catalytic flavoprotein domain. Substrate binding may be assisted by the adjacent domains, e.g., the CH and LIM domains.

An important contribution to the understanding of MICAL and, therefore, of semaphorin signaling and of the control of cytoskeleton dynamics may be given by in depth *in vitro* biochemical studies of MICAL forms. These studies are still limited, and will be reviewed in this article.

### 2.3. Distribution of MICAL Forms

Since the pioneering work of Suzuki *et al*. [1] and Terman *et al*. [2] it has become clear that MICAL forms are encoded in the genomes of several animals, from insects to humans. In databanks, MICALs are also classified as NEDD9-interacting protein (with NEDD9 being a synonym of CasL) or “CH and LIM containing proteins”. Recently, the misleading term “methionine sulfoxide oxidase” has been added alluding to the recent proposal that Drosophila MICAL specifically converts actin Met44 side chain to methionine sulfoxide [52].

**Figure 4:**
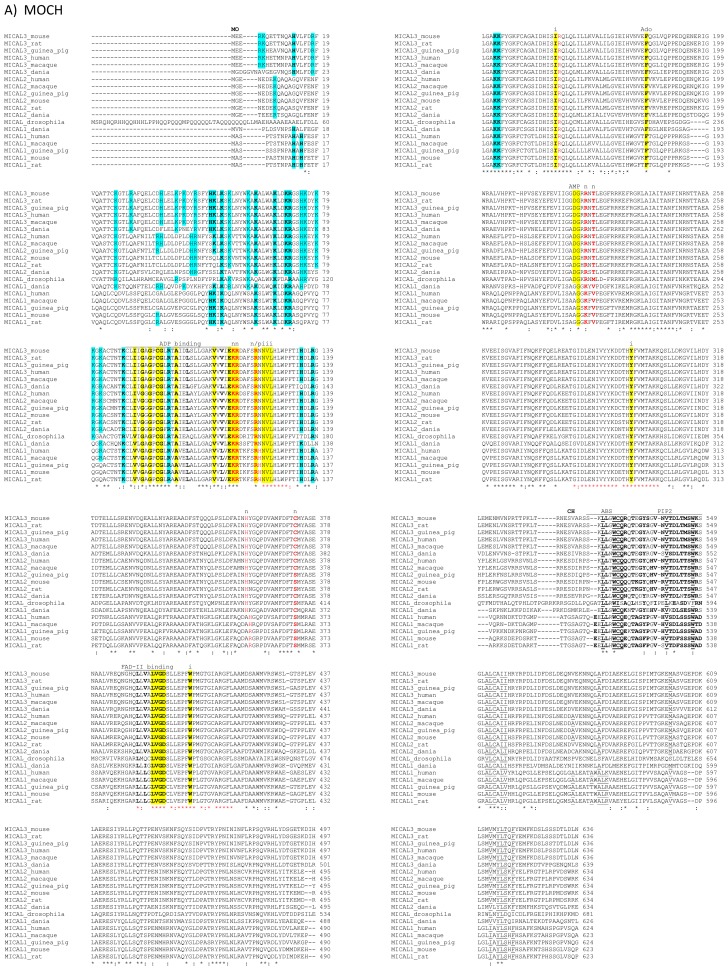

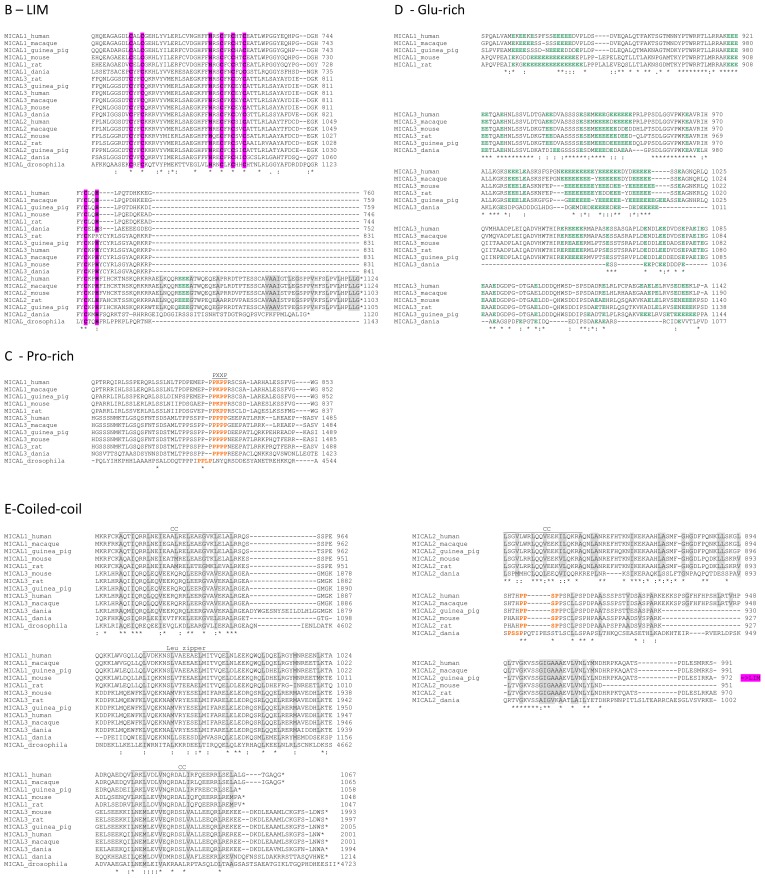
Sequence comparison of MICAL forms. Regions corresponding to the monooxygenase (MO) and calponin homology (CH) domains (**A**), LIM domain (**B**), Pro-rich (**C**), Glu-rich (**D**) and *C*-terminal coiled-coil regions (**E**) of selected MICAL sequences (Table 1) have been aligned with ClustalW2 [61]. The output reflects the degree of sequence similarity among the proteins. The sequences were annotated as follows, with the numbering of mouse MICAL1. **A**: cyan, basic residues in the *N*-terminal proteins regions forming one of the patches of basic potential on MICAL-MO. In bold are conserved residues identified by Nadella *et al*. [4] and those identified by us from sequence and structural comparisons. Yellow, residues implicated in FAD binding as specified above the sequences. The residues matching the ADP binding region identified as in [62] and those matching the second FAD consensus sequence identified based on [63] are in bold; the additional residues that interact with the isoalloxazine ring (i), the pyrophosphate (p), adenosine (Ado) and AMP regions of FAD, as identified in [3], are also indicated. Red, residues proposed to be involved in the interaction with NADPH as deduced from the comparison with PHBH [64] and structural analysis [3,4]. In bold are residues indicated by [3]. In plain characters are those identified by us as also contributing to the formation of the patch of basic potential for NADPH binding. The conserved regions of MICAL’s MO domain are indicated with the red stars below the aligned sequences as in [3]. In the CH domain, residues forming the hydrophobic core as based on the structure of the CH domain of human MICAL1 (PDB ID 2DK9, [65–67]) are underlined. The regions corresponding to the actin binding site (ABS) of type 2 calponin homology (CH2) domains and to the phosphatidylinositol (4,5)-bisphosphate (PIP2) binding site also found in type 2 CH domains are also indicated. Residues matching the consensus sequence for the formation of the ABS and PIP2 binding sites, as identified in [65,66], are in bold; **B**: in the LIM domain, the conserved C683, 686, 707, 710, 713, 733 and H704, H736 residues proposed to coordinate the zinc ions in the zinc-finger motifs according to reference [18] are bold and purple. In MICAL2 sequences, the *C*-terminal protein region next to the LIM domain is included; **C**: the Pro-rich regions of MICAL1 and 3 matching the PXXP consensus sequence for the formation of a SH3 binding domain initially identified in MICAL by Suzuki *et al*. [1] are in orange. In MICAL2 a sequence matching the PXXP motif is found before the LIM domain and is shown in orange in panel E; **D**: the Glu-rich regions found in MICAL1 and 3 are aligned separately with the Glu residues in green. A Glu-rich region is highlighted green in the *C*-terminal region of MICAL2 in B; **E**: the regions potentially forming coiled-coils (CC) are aligned for MICAL 1 and 3 and for MICAL2. The hydrophobic residues of the potential heptad repeats are colored grey. The region potentially forming a leucine zipper in MICAL1 and 3, as identified in [1], is indicated. In MICAL2, the LIM domain (**B**) is after the putative coiled-coil region, and is followed by a region where a coiled-coil structure may also be formed (see **B**).*, identical residues; :, conserved residues.

Insects contain only one gene encoding MICAL, which, in Drosophila, leads to several MICAL isoforms through alternative splicing [8,9]. The model organism zebrafish (*Danio rerio*) contains three different genes encoding MICAL isoforms [40], as well as several MICAL-like species. Mammals also have three genes for MICAL, which encode MICAL1, MICAL2 and MICAL3 (Table 1) beside several genes for MICAL-like proteins. The MICAL1-3 proteins are clearly related to each other. The region spanning the *N*-terminal flavoprotein and CH domains is conserved across all MICAL isoforms. On the contrary the location of the LIM domain and of the regions possibly containing coiled-coil motifs differ (Figures 1 and 4) for the length of the (poorly conserved) interdomain regions, their arrangement in the protein and the degree of sequence conservation. Analysis of transcripts in various mammalian tissues predicts that each MICAL-encoding gene may lead to the production of different protein forms through alternative splicing events. Some of these alternative species may lack one or more of the protein domains or parts of them and it is still unknown if they are biologically relevant [3,4,9,20,25,59]. Exceptions are the splicing variants PVa and PVb of MICAL2 discovered by Ashida *et al*. [37], which have been detected at the protein level, as discussed below. Different genes encode the group of MICAL-like (MICALL) proteins, which lack the *N*-terminal flavoprotein region and show different arrangements of the CH, LIM, and coiled-coil domains [44,46]. MICALL2 is also known as JRAB. It is a Rab13 effector protein and appears to function as a scaffold protein participating in endocytic recycling, the formation of functional tight junctions and the scattering of epithelial cells (as reviewed in [60]).

A bioinformatic analysis restricted to human proteins led to establish phylogenetic relations among the so-called class of CH and LIM-containing proteins, which includes MICAL1-3 as well as the LMO7 and LIMCH1 gene products, highlighting a possible common evolutionary origin of these proteins [59]. The latter proteins are also involved in interactions with actin and actin-binding proteins.

No genetic mutations of MICAL have been identified in humans, yet. However, a specific link between MICAL and disease was found by Ashida *et al*. [37] who identified the human MICAL2 splicing variants PV1a and PV1b mentioned before. These proteins are specifically highly expressed in the cytoplasm of cancer cells of particularly aggressive and difficult to treat prostate cancer forms. Interestingly, MICAL2 prostate variants PVa (AB126828, 6805 b, 976 residues, 112 kDa) and PVb (AB126829, 6742 b, 955 residues, 109 kDa) apparently derive from alternative splicing of MICAL2 transcript (EST sequence AF052170, cDNA sequence NM_014632, 3885 b, 126 kDa) leading to predicted protein forms that are similar to each other and to MICAL2 in the flavoprotein, CH and LIM domains, but differ in the *C*-terminal region. The consequences of such mutations on semaphorin-signaling and interactions with components of the pathway downstream of the semaphorin-plexin interaction have not been defined yet. However, it has been shown that lowering the expression of these forms by small interfering RNA resulted in drastic growth suppression in cancer cell lines.

Genetic studies in *Drosophila* associated the milch (or milchstrasse) phenotype with point mutations or deletion of extended chromosomal regions including the single MICAL gene [2,23]. These mutants have been used as the genetic background for several studies aimed to understand MICAL function [2,23,52,53].

### 2.4. Analysis of MICAL Primary Structure

The primary structures of representative MICAL forms are compared in Figure 4, which has also been annotated on the basis of information deriving from structural studies [3,4,65]. In the following text and in Figure 4 the residue numbering is given for mouse MICAL1, for which high resolution structural models are available [3,4].

In the *N*-terminal part of the protein the presence of a flavoprotein domain was initially hypothesized on the basis of the presence of regions matching the consensus sequence for the formation of a Rossman fold for the binding of the ADP moiety of FAD or pyridine nucleotides (residues 87–114, [62]) and of the so-called second FAD consensus sequence identified by Eggink *et al*. (residues 383–393, Figure 4, [63,69]).

**Figure 5:**
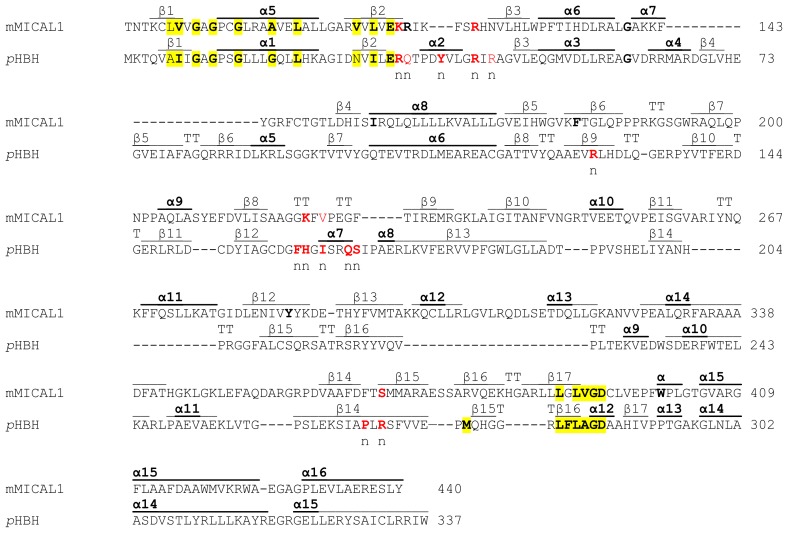
Comparison between mouse MICAL and p-hydroxybenzoate hydroxylase. The 83–440 region of mouse MICAL1 is compared with full-length *Pseudomonas fluorescens p*-hydroxybenzoate hydroxylase. The alignment takes into account the known three-dimensional structures of the proteins (2BRA [4], 2BRY [3] for mouse MICAL; 1PBE [68] for PHBH) as in [3,4]. The secondary structure elements (β strands, α helices and β turns (TT) are indicated above each sequence. The conserved residues in the consensus sequence for the formation of the binding site of the adenylate portion of FAD [62] and the second FAD consensus sequence of Eggink *et al*. [63] are colored yellow. The residues that in PHBH are implicated in NADPH binding [64] are indicated in red and with “n”. If they are conserved in equivalent positions in all MICALs (see Figure 4) they are in red in both sequences. Ser368 (conserved as S or C in all MICAL sequences) proposed to be equivalent to Arg269 of PHBH [3] is also indicated.

An overall sequence similarity with proteins of the *p*-hydroxybenzoate hydroxylase (PHBH) structural family ([5,6] and references therein) was found beyond the first ≈80 residues of MICALs leading to the hypothesis of the flavoprotein nature of its ≈500 residues *N*-terminal domain (Figures 4 and 5). This hypothesis was later confirmed by the determination of the three dimensional structure of mouse MICAL1 *N*-terminal region [3,4] and by *in vitro* studies on the corresponding region of mouse [4] and human [70] MICAL1.

In the *N*-terminal segment (~80 residues), some residues are conserved in all MICALs. Other residues are only conserved within each MICAL 1–3 class and may be diagnostic for the classification of MICAL forms. *Drosophila* MICAL contains an ≈40 residues *N*-terminal extension with respect to mammalian MICALs. However, it is not a distinctive feature of insects MICALs, for which most of the sequences in databanks are not complete.

The flavoprotein-like domain is followed by a calponin homology (CH) domain (residues 506–612, Figures 1 and 4, [65]). Sequence (Figure 4) and structural analysis [65] identified it as a type 2 CH domain (see below). The LIM domain (residues 673–741), which forms zinc finger structures [17,18], is present in one copy in all MICAL forms with the conserved Cys and His residues implicated in zinc ions coordination. However, in MICAL2 the region separating it from the CH domain is longer than in MICAL1 and 3 (Figures 1 and 4). Furthermore, it is between a possible coiled-coil motif and the *C*-terminus, which may also form a coiled-coil structure. In contrast, such coiled-coil motifs are only *C*-terminal to the LIM domain in MICAL1 and 3.

In the *C*-terminal region, a Pro-rich region matching the PXXP motif typical of proteins that interact with SH3 domains is found in MICAL1 [1] and MICAL3 only (Figure 4C). However, a Pro-rich region possibly matching the PXXP consensus sequence is actually found also in MICAL2, but in the region containing the putative coiled-coil motifs (Figure 4E). MICAL1 and 3 exhibit Glu (and Asp)-rich regions of different length (Figure 4D). They may also contribute to protein-protein interactions, but their precise role in MICALs is not known. In MICAL2, a Glu-rich region is found in the *C*-terminal protein segment following the LIM domain, but it is very short and is unlikely to play a specific role (Figure 4B).

The final part of MICAL1 and 3 may form coiled-coil structures similar to that found in the α domain of proteins of the Ezrin, Radizin and Moesin (ERM) family as pointed out by Terman *et al*. [2]. The number and distribution of heptad repeats vary among MICALs (Figure 4E). In MICAL1, one of the coiled-coil motifs (residues 964–986) corresponds to the leucine zipper identified by Suzuki *et al*. [1].

The last four residues of MICAL have been suggested to match the consensus sequence (S/TXV) for the formation of a PDZ binding motif [2,23], but comparison of multiple sequences does not allow us to confirm the hypothesis (Figure 4E).

### 2.5. High Resolution Structure of MICAL Forms

A breakthrough in the understanding of the possible function of MICAL was the determination of the high resolution structure of the *N*-terminal flavoprotein-like region of mouse MICAL1 by two groups [3,4]. Confirmation of the presence of a CH and of a LIM domain in MICALs was obtained by nuclear magnetic resonance (NMR, see below). The 1–489 region of mouse MICAL1 was produced in *E. coli* cells in fusion with an *N*-terminal His_6_-tag [3,4]. Well-diffracting crystals were obtained by Siebold *et al*. [3] using such a protein form, while the Nadella *et al*. [4] crystals were obtained using an engineered protein form, which carried the Lys-to-Ala double substitution of surface residues 141 and 142. As expected, the structures of the as-isolated (oxidized) proteins (2 Å resolution, PDB ID 2BRA for [4]; 1.45 Å resolution, 2BRY for [3]) are similar to each other.

A homodimer was found in the crystallographic asymmetric unit, although the protein is monomeric in solution. Only a few disordered segments are present so that the details of the structure can be examined. Each monomer contains one FAD coenzyme and shows an *N*-terminal four-helix bundle region (residues 1–85) followed by a core region (residues 86–442) and a *C*-terminal segment (residues 443–489).

The core region exhibits the PHBH fold, which is typical of several FAD-containing oxidases and monooxygenases. In particular the highest structural similarity is with PHBH (1PBE) and phenol hydroxylase (1PN0). Thus, the core of MICAL flavoprotein domain is bilobal in shape. The larger portion contains the FAD binding region and is formed by residues 86–234 and 367–444 (the boundaries of domains are as described in [3] and are, as expected, similar to those described in [4]). This region should also contain the NAD(P) binding site of MICAL by analogy with the enzymes of the PHBH class (see below). The region between residues 235 and 366 forms the smaller lobe of the protein, which completes the active site. This 235–366 region was defined as the monooxygenase domain by Siebold *et al*. [3], but we will indicate it as the substrate binding domain in order to adopt the nomenclature used for PHBH [68,71].

**Figure 6:**
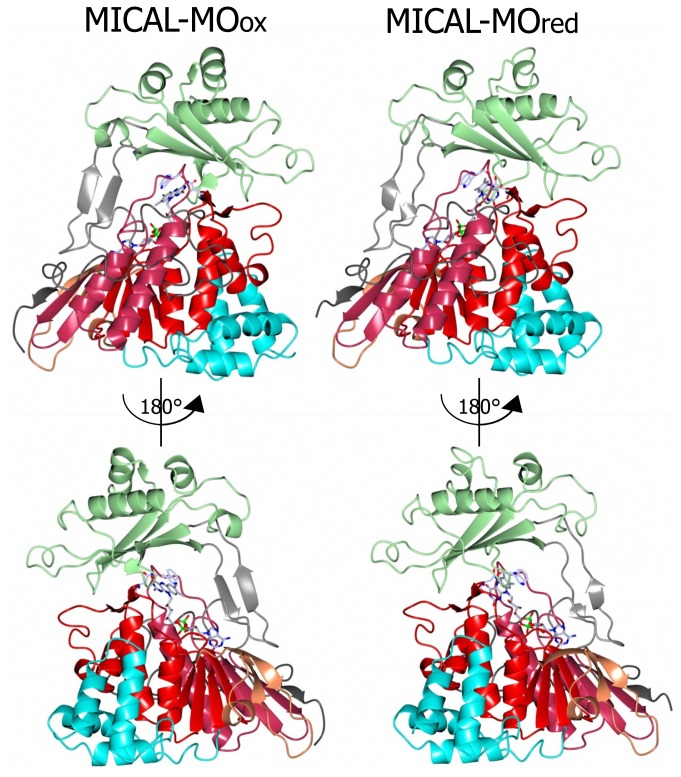
Comparison of the structures of MICAL1 monooxygenase domain. The structures of the “as isolated” mouse MICAL1 monooxygenase domain in the oxidized (“flavin out”) state (2BRY, chain A, MICAL-MO_ox_) and of the NADPH-reduced (“flavin in”) species (2C4C, chain A, MICAL-MO_red_) as determined by Siebold *et al*. [3] are shown in two orientations. Color scheme is as follows: blue, residues 1–85; red, residues 86–177, 208–217; coral, 178–208; grey, 218–237 (β9) and 366–375 (β15); green, 238–365; dark red, 376–442; dark grey, 443–489. FAD, Trp400 and Asn123 are shown in sticks with carbon in light grey, nitrogen in blue, oxygen atom in red and phosphorus in green. The CCP4MG program was used to generate the figure [72].

The flavoprotein domain of mouse MICAL1 shows a simplified topology with respect to PHBH, which may allow for a degree of flexibility higher than in PHBH. In PHBH the polypeptide chain passes several times between the FAD and the substrate binding domains. In MICAL, the FAD binding region and the substrate binding domain are only connected by a long two-stranded β sheet formed by β strands β9 and β15 (Figures 5 and 6). Such a difference is due to the absence, in MICAL, of regions corresponding to PHBH β5 and β6 strands and α4 helix (Figure 5), which are replaced by α-helix 11 of MICAL’s substrate binding domain.

Both the *N*-terminal 1–85 region and the *C*-terminal 442–489 segment are ordered and make extensive contacts with the core region. Not surprisingly, the *C*-terminal 443–489 region of MICAL, which connects the flavoprotein domain to the CH domain, has no similarity with PHBH *C*-terminal segment, which forms the interface domain stabilizing the functional homodimer.

**Figure 7:**
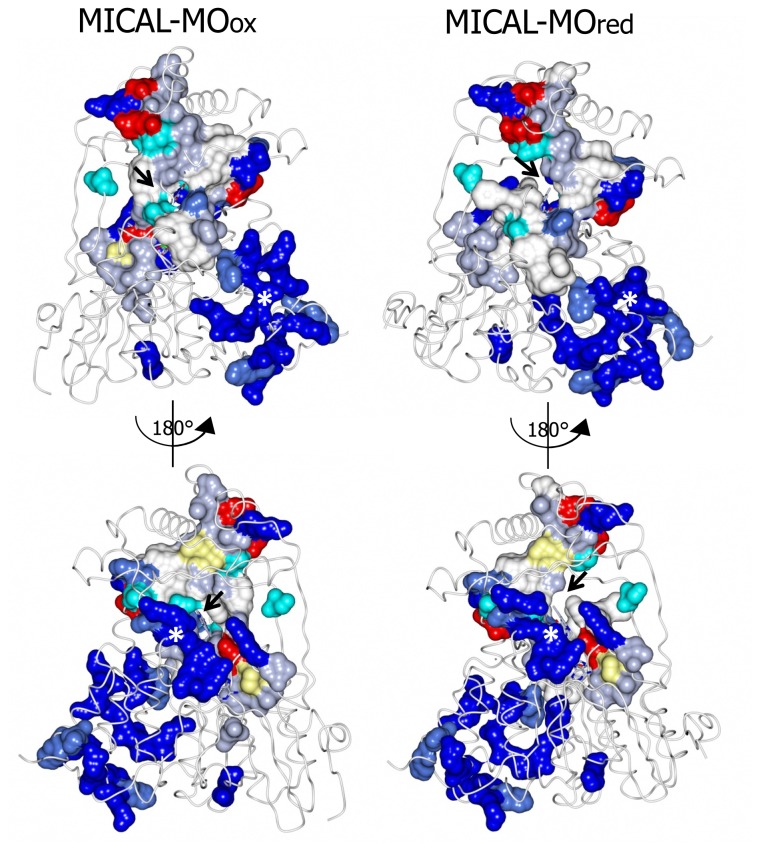
Comparison of the “flavin out” (MICAL-MO_ox_) and “flavin in” (MICAL-MO_red_) conformations of MICAL-MO highlighting the conserved residues forming the putative NADPH binding site and the tunnel leading to the active site in the “flavin in” conformation. The stars mark the positively charged residues (in spheres) in the *N*-terminal protein region (top panels) and those in the putative NADPH binding site (lower panels; see Figures 4 and 5). The residues in the conserved regions surrounding the FAD isoalloxazine ring (arrows) and comprising residues 121–130, 278–300 and 390–405 (red stars in Figure 4) are shown. Color code is: light grey, Phe, Tyr, Trp; grey, Gly, Ala, Val, Leu, Ile; cyan, Ser, Thr, Gln, Asn; light yellow, Cys, Met; blue, Arg, Lys, His; red, Asp, Glu. The view is similar to that in Figure 6 to highlight the formation of the tunnel leading from the protein surface to the isoalloxazine ring in the “flavin in” (right) conformation. The CCP4MG program was used to generate the figure [72].

**Figure 8:**
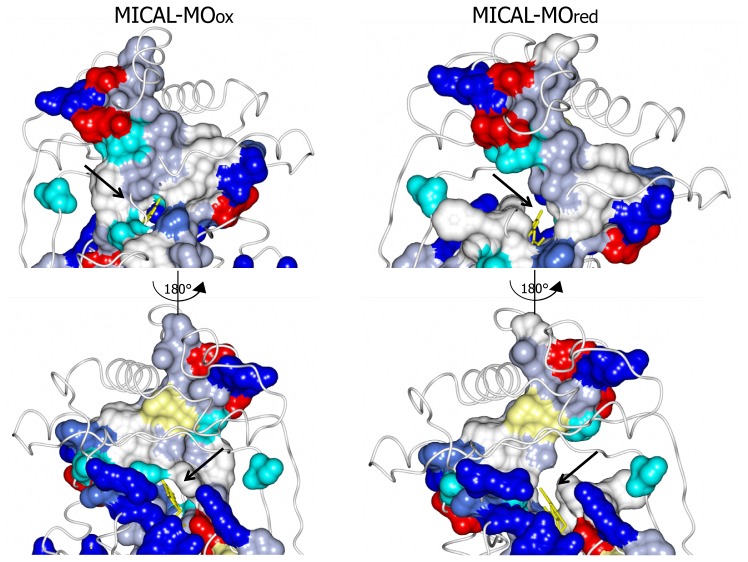
Details of the “flavin out” (MICAL-MO_ox_) and “flavin in” (MICAL-MO_red_) conformations of MICAL-MO highlighting the conserved residues forming the tunnel leading to the active site in the “flavin in” conformation. The residues in the conserved regions surrounding the FAD isoalloxazine ring (arrows) and comprising residues 121–130, 278–300 and 390–405 (red stars in Figure 4) are shown. The views correspond to those shown in Figure 7. Color code is also as in Figure 7 except for FAD that is in sticks and yellow. The CCP4MG program was used to generate the figure [72].

The *N*-terminal 1–85 region, which has no counterpart in PHBH, contains several basic residues. Some of them are conserved across all MICALs or are found in most MICAL sequences (H11, H13, H49, K50, K52, K61, K66, K(or R) 69, R(or K)70, K(or R)86, Figures 4 and 7). These residues, as well as several conserved residues in the initial part of the FAD domain (R98, K142, K141, R136, H133, Figures 4 and 7), contribute to different extents to the formation of one extended patch of basic potential found on one face of the protein (Figures 7 and 8). Such a positively charged region is on the same side of the protein as several strictly conserved segments of MICALs (residues 121–130, 278–300, 390–411), which surround the FAD isoalloxazine ring and may contribute to the definition of MICAL (protein) substrate binding region and catalytic site. Due to its location and to the acidity of several cytoskeletal proteins, including actin, the *N*-terminal 1–85 basic region of MICAL has been proposed to be important to promote the interaction with the putative protein substrate [3,4].

A second patch of basic potential is found on the opposite face of the protein (Figures 7 and 8; [3,4]). It is formed by the MICAL’s conserved residues K115, R116, R121, H122, R158, K221, R356 (Figure 4). Sequence (Figure 5) and structural comparison between MICAL and PHBH indicates that these residues may contribute to NADPH binding. Determination of the structure of the R220Q variant of PHBH (with the flavin in the reduced form) in complex with NADP(H) ([64], PDB ID 1K0J) showed that NADPH binds in an extended conformation on the protein surface in a groove that crosses the FAD binding site and is lined by several protein side-chains and by the pyrophosphate and adenosyl moieties of FAD. Residues K115, R116, R121, K221, V223 and S368 of MICAL may correspond to R33, Q34, R42, H162, R269 and I164 of PHBH, which participate in NADP(H) binding ([4,64], Figure 5).

As in enzymes of the PHBH structural family, FAD is bound to MICAL in an extended conformation. Its adenylate moiety is bound to the Rossman fold identified by the GXGXXG motif at positions 91–96 [62,68,71]. A β strand (β17, [4]) formed by residues 384–390 interacts with the ribityl 3′OH and corresponds to the Eggink’s second FAD consensus sequence [63] detected in MICAL’s primary structure (Figure 4A). Both the dimethylbenzene and the pyrimidine rings of FAD are exposed to solvent (Figures 6, 7, 8) on the two opposite faces of the protein. This is at variance with PHBH in which the dimethylbenzene ring of the flavin points towards the solvent where NADPH binds, while the pyrimidine ring is buried within the protein. Several of the residues interacting with FAD isoalloxazine ring belong to the highly conserved MICAL’s regions (see above).

**Figure 9:**
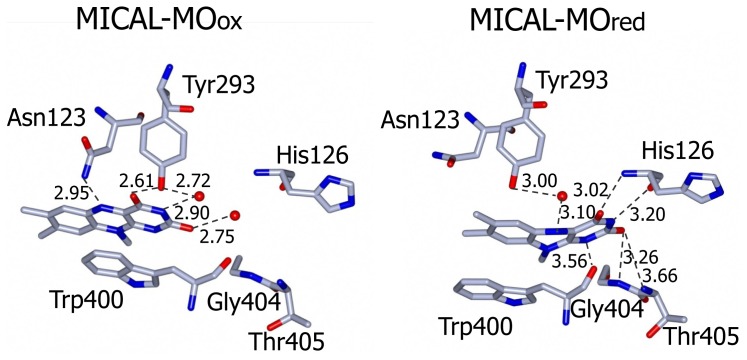
Details of the FAD environment in the “flavin out” and “flavin in” conformations of MICAL-MO. The figure is drawn based on [3]. The view is obtained by vertically flipping the models in Figure 6 with an approximately 45° rotation. The FAD ribityl side chain is cut at C1′. The CCP4MG program was used to generate the figure [72].

The side-chain of the conserved Trp400 stacks against the *re* face of the flavin isoalloxazine ring and must undergo conformational changes in order to allow the productive binding of the nicotinamide portion of NADPH during the enzyme reductive half reaction (see below). On the isoalloxazine *si* side, van der Walls interactions are made with Ile157 side chain. Asn123 side-chain amide is at hydrogen bonding distance from the flavin N5 position and is held in place by a complex network of hydrogen bonding interactions between the amide oxygen atom and Asn243, Thr291 and Asp360. The side-chain hydroxyl group of Tyr293 is hydrogen bonded to FAD O(4) contributing to the stabilization of the observed conformation. The FAD N(1), O(2) and N(3) atoms interact with water molecules. Wat71 is held in place by Tyr293 and Val124, while Wat 95 may interact with Asp 393 side-chain carboxylate and with the ribityl 2′OH group (Figure 9).

In support of the hypothesis that MICAL is a monooxygenase of the PHBH class is the fact that the conformation of the flavin cofactor corresponds to the “flavin out” conformation of PHBH. Such a “flavin out” conformation is observed only with some mutant forms of PHBH or in the presence of the alternative substrate 2,4 dihydroxybenzoate (see [5,6] and references therein), while, in most cases, PHBH is in the “flavin in” state.

Incubation of MICAL crystals with NADPH led to the determination of the structure of the protein with FAD in the reduced form (as deduced from the bent conformation of the isoalloxazine ring) in a conformation corresponding to the “flavin in” state of PHBH (PDB ID: 2C4C, chain A, [3], Figures 6, 7, 8, 9). No NADP(H) was bound to the protein preventing the identification of its binding site. In one of the chains (chain B) both “flavin out” and “flavin in” conformations are observed.

The switch of FAD from the “out” to the “in” conformation occurred through a rotation (~20°) of the ribityl C1–C2 bond, which was accompanied by domain reorientation. In the “in” conformation the isoalloxazine ring is shielded from solvent and buried at the interface between the flavin and the substrate binding domain (compare left and right panels of Figure 8).

The three water molecules interacting with the flavin N(1), O(2), N(3) and the ribityl 2′OH are displaced with their position occupied by the FAD pyrimidine ring, which now interacts with His126 main chain amide and carbonyl groups, Gly404 and Thr405 (Figure 9). Tyr293 side chain hydroxyl group now interacts through a water molecule with the FAD N(5) position. The ring stacking interaction between the flavin isoalloxazine ring and Trp400 side-chain is lost, but Trp400 main chain oxygen atom is at hydrogen bonding distance from the reduced FAD N(5) atom (Figure 9). It has been proposed [3] that the loss of hydrogen bonding interaction between Tyr293 of the substrate binding domain and FAD O(4) upon flavin reduction may trigger the conformational switch from the “flavin out” to the “flavin in” state. The latter is accompanied by conformational changes of the polypeptide chain with changes in secondary structure elements in the substrate binding domain (compare Figures 6 and 7 left and right panels). The 6.5° difference in the relative position of the FAD and substrate binding domains of oxidized and reduced MICAL is larger than that observed in PHBH due to the simpler topology of MICAL as compared to PHBH. The β sheet formed by β9 and β15 connecting the FAD and the substrate binding domain has been proposed to act as a hinge during domains reorientation [3].

The MICAL region corresponding to the substrate binding cavity of PHBH shows no similarity to PHBH, regardless of the protein conformations selected for comparison [3,4]. On the contrary, comparison of the structures of MICAL in the oxidized (“flavin out”) and reduced (“flavin in”) forms reveals how the hydroxylatable substrate may access the reduced flavin [3]. The domain reorientation that accompanies the switch from the “flavin out” to the “flavin in” conformation leads to the formation of a channel connecting the protein surface to the active site above the flavin isoalloxazine ring (highlighted in Figure 8). In this conformation the central ring of FAD, with the reactive C4a-N5 position, becomes solvent accessible. The residues lining this channel are conserved in MICALs suggesting an important functional role (Figure 4). The channel, which appears to be rather hydrophobic, is roughly located on the side of the protein where the positively charged N-terminal domain unique to MICAL lies.

These observations, together with the presence of typical protein interaction domains in full-length MICAL, support the hypothesis that the *N*-terminal flavoprotein-like domain catalyzes a monooxygenase reaction and that the substrate may be a protein side-chain. The *N*-terminal positively charged region of the MICAL monooxygenase domain may contribute to the binding and orientation of the protein substrate with the side-chain to be modified accessing the catalytic site through the tunnel. The MICAL’s additional domains may affect substrates binding as well as the conformational changes that are likely to take place during the catalytic cycle of MICAL, by analogy with PHBH mechanism ([5,6] and references therein). Starting from the oxidized (“flavin out”) state, binding of NADPH should trigger a first conformational change involving reorientation of Trp400 side-chain and allowing correct positioning of the NAD(P)H nicotinamide ring for hydride transfer to the flavin N(5). Next the protein should switch to the “flavin in” conformation in which reduced FAD can react with molecular oxygen to generate the 4a-hydroperoxide intermediate in a protected environment that would allow hydroxyl transfer to the (protein) substrate and release of a water molecule rather than of hydrogen peroxide or superoxide (Figure 3). When the MICAL (protein) substrate would come into play in the catalytic cycle cannot be predicted from structural analyses. PHBH is virtually inactive in the absence of the substrate to be hydroxylated, exhibiting a very low NADPH oxidase activity. Although NADPH can bind to the free enzyme, it can reduce the flavin coenzyme (in the “flavin out” state) only when the hydroxylatable substrate is present [5,6]. In PHBH, NADP^+^ is released prior to the switch to the “in” conformation so that no contribution to the control of oxygen reactivity is made by the oxidized nicotinamide product. This is at variance with the role of NAD(P)^+^ in the catalytic cycle of another important class of monooxygenases exemplified by the flavin monooxygenase (FMO) from *Methylophaga sp*. strain SK1 [73]. In this class of monooxygenases, bound NADP^+^ contributes to promote the hydroxylation reaction [74–78].

The structures of the CH domain of human MICAL1 (PDB ID: 2DK9, [65,79]; PDB ID: 1WYL, no accompanying paper), MICAL2 (PDB ID: 2E9K, no accompanying publication) and MICAL3 (PDB ID:2D88, no accompanying paper) and of the LIM domain of human MICAL1 (residues 687–755, PDB ID 2CO8; no accompanying publication) were determined by NMR confirming their sequence-based identification (Figure 4A,B). These studies also demonstrated that it is in principle feasible to produce these protein fragments and, e.g., to study their ability to interact with MICAL interactors or the MICAL monooxygenase domain (and other truncated forms) and how they modulate MICAL’s catalytic properties.

Calponin homology (CH) domains are protein interacting domains that are often found in actin and microtubule binding proteins [66,67,80,81]. In most of the F-actin binding or bundling proteins two tandem CH domains are present and form the actin binding site. MICALs contain a single type 2 CH domain ([59,65], Figure 4), which is predicted not to be sufficient to bind F-actin [80,81]. Accordingly, no binding between the isolated MICAL1 CH domain and F-actin could be detected [65]. Thus, it has been proposed that the CH domain may assist regions of the MICAL’s monooxygenase domain in (protein) substrate binding either by completing the binding site, or by establishing the initial interaction with the protein substrate and presenting it to the catalytic domain. In this respect, the work of Hung *et al*. [52,53], which will be discussed below, demonstrated for the first time that the MICAL monooxygenase-like domain is sufficient to interact with F-actin causing its depolymerization in a NADPH-dependent reaction.

LIM domains (from the initials of the homeodomain proteins Lin11, Isl-1 and Mec-3, [17,18]) are cysteine- and histidine-rich, zinc-coordinating domains, which contain two tandemly repeated zinc fingers. The LIM domain is a protein interaction module found in structural proteins, kinases and transcription factors with roles in gene expression, cell adhesion and signal transduction [17,18]. No information on its precise role in MICAL is yet known.

### 2.6. Catalytic Activities of MICAL

*In vitro* mechanistic studies of MICAL forms are very much needed to complement genetic and in cell studies as well as structural work. However, in spite of the fact that the protein was first identified in 2002 [1,2] and structures of the monooxygenase domain are available since December 2005 [3,4], very little mechanistic work has been done on isolated protein forms. However, essential key observations have been reported recently using mouse [4], human [70] and Drosophila MICAL forms [52,53]. The mouse MICAL1 monooxygenase domain has been produced by Siebold *et al*. [3] and Nadella *et al*. [4] with the aim to solve its three-dimensional structure by X-ray crystallography. As a complement to the structural studies, Nadella *et al*. [4] reported some kinetic properties of the mouse MICAL1 monooxygenase domain, which we shall here indicate as MICAL-MO or simply MO.

A coupled assay with horse radish peroxidase (HRP) and amplex red was used to determine that mouse MICAL-MO catalyzes the oxidation of NAD(P)H with production of hydrogen peroxide. The enzyme was reported to prefer NADPH over NADH as the reductant and to exhibit a *k*_cat_ of 77 s^−1^ and a *K*_M_ for NADPH of 222 μM at pH 7.0, which was indicated as the pH optimum for the activity. (−) epigallocatechin gallate (EGCG) was reported to be a potent inhibitor of mouse MICAL-MO domain (*K*_i_, 2 μM), non-competitive with respect to NADPH. This result is very interesting in the light of previous work that demonstrated that treatment of dorsal ganglion cells with EGCG mimics MICAL inactivation by making cells insensitive to semaphorins [2,38]. Furthermore, xanthofulvin, a natural compound structurally related to EGCG, was also found to inhibit semaphorin signaling and to promote neuronal regeneration following spinal cord injury [82,83]. Forms of the Drosophila MICAL comprising the monooxygenase domain (MICAL-MO) or both the monooxygenase and the CH domain (MICAL-MOCH) have been produced as fusions with *N*- or *C*-terminal Nus and His tags [20,52,53]. The purified proteins have been mainly used to study their interaction and effect on actin [52,53], but no information is available on flavin content and spectral properties of the proteins, nor on the kinetics of the catalyzed reactions.

More recently, the monooxygenase domain of human MICAL1 was produced in *E. coli* cells without or with *N*- or *C*-terminal His-tags [70]. The purified proteins contained stoichiometric amounts of FAD and were indistinguishable from each other with respect to the absorbance and fluorescence spectra and the steady-state kinetic parameters of the NAD(P)H oxidase reaction. Thus, most of the experiments designed to carry out the basic characterization of the protein have been carried out with the most abundant *C*-terminally His-tagged MICAL-MO form.

**Figure 10:**
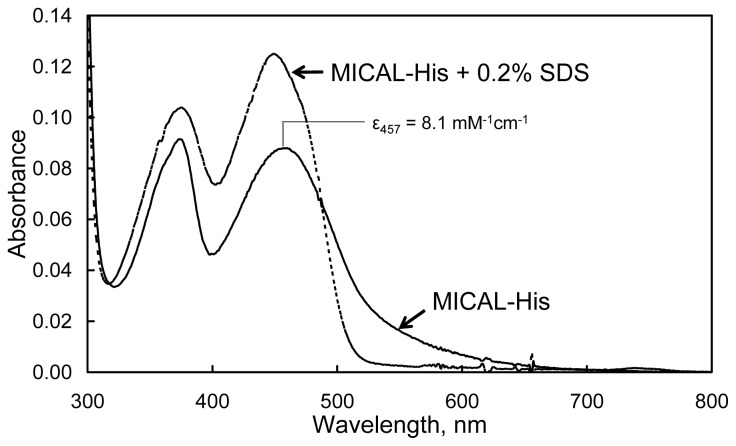
Comparison of the absorption spectra of human MICAL-MO and of the FAD cofactor released after protein denaturation. Reprinted from [70] with permission from Elsevier.

The absorbance spectrum of human MICAL-MO exhibits maxima at 278, 376 and 457 nm with a *A*_278_/*A*_457_ ratio of ~13. The calculated extinction coefficient at 457 nm was 8.1 mM^−1^ cm^−1^ (Figure 10). The broad absorbance band extending to 700 nm agrees well with the presence of Trp400 side-chain, which is positioned essentially parallel to the FAD isoalloxazine ring in the structure of the “as isolated” (oxidized) mouse MICAL-MO at a distance suitable for the formation of a charge-transfer complex (Figure 9, [3,4]). Such an absorption band might provide a tool to monitor the conformational changes that are predicted to take place during the catalytic cycle of MICAL-MO. The FAD bound to human MICAL-MO does not interact with sulfite nor stabilizes 1-electron reduced semiquinone forms of the flavin during photoreduction in the presence of EDTA and deazariboflavin, chemical reduction with dithionite or NADPH or exposure to air of the reduced enzyme. From anaerobic NADPH titration at pH 7.0 a midpoint potential of ≈ −150 mV was calculated for the *E*_ox_/*E*_red_ couple. These properties support the functional similarity of MICAL-MO with enzymes of the FAD-dependent monooxygenase family whose prototype is PHBH, rather than typical FAD-dependent oxidases [55,84], which also exhibit the PHBH fold (e.g., d-amino acid oxidase; [85]). No spectral changes were observed by titrating the enzyme with NADP^+^, a fact that may limit the study of the interaction between MICAL-MO and the pyridine nucleotide substrate/product in future work.

As in the case of the mouse protein, human MICAL-MO catalyzes NAD(P)H oxidation in the presence of atmospheric oxygen with a preference for NADPH over NADH in terms of both *k*_cat_/*K*_NAD(P)H_ and *k*_cat_ (Table 2). By comparing the rate and extent of NADPH oxidation (spectrophotometrically) and of oxygen consumption (with an oxygen electrode), in the presence and absence of catalase and/or superoxide dismutase, it was concluded that one NADPH is oxidized to NADP^+^ per molecule of oxygen being reduced to hydrogen peroxide.

The 1:1 stoichiometry between oxidized NADPH and hydrogen peroxide being produced was confirmed by quantifying the hydrogen peroxide product with HRP and amplex red or *o*-dianisidine. Interestingly, it was found that the NADPH oxidase activity of MICAL-MO could not be measured in a coupled assay with HRP and amplex red or *o*-dianisidine due to interference of NADPH with the HRP reaction and a stimulation of NADPH oxidation by HRP and amplex red in the presence of H_2_O_2_. Thus, H_2_O_2_ production by MICAL-MO NADPH oxidase activity could only be reliably quantified in a two-step assay in which NADPH was allowed to be oxidized by MICAL-MO, the reaction was stopped by rapidly oxidizing residual NADPH (with glutamate synthase and its substrates as a suitable dehydrogenase, [87]), and *o*-dianisidine and HRP were eventually added to quantify H_2_O_2_.

Both the *k*_cat_ (~4 s^−1^) and the K_M_ for NADPH (20–30 μM) were one order of magnitude lower than the values reported for the mouse species (77 s^−1^ and 222 μM, respectively, [4]). The differences in the *K*_M_ values for NADPH is due to the presence of 0.1 M KCl in the assays done with the mouse enzyme and to the sensitivity of MICAL-MO to the ionic strength of the medium and the type of anions (see Table 2 and below). In the light of the strong sequence similarity between the mouse and human proteins, the different *k*_cat_ values are more likely due to a combination of the assay methods adopted (direct measurement of NADPH oxidation for the human protein versus the coupled HRP/amplex red assay for the mouse protein) and other experimental factors, rather than actual species-dependent differences. The NADPH oxidase activity of human MICAL-MO is significantly higher than that of PHBH, taken as the reference enzyme. The latter is virtually inactive in the absence of the *p*-hydroxybenzoate substrate [5,6]. Whether the MICAL-MO NADPH oxidase activity is physiologically relevant is unclear. MICAL-MO NADPH oxidase activity is very sensitive to ionic strength and type of anions (see below) so that it is expected that the reaction is very slow in the cell.

However, whether such a sensitivity is removed by the other MICAL’s domains and the interacting proteins will need to be established. *In vitro*, the detectable, NADPH oxidase activity of MICAL-MO provides a tool to study, perhaps for the first time, the reaction between a monooxygenase of the PHBH class and NADPH in the absence of the substrate undergoing hydroxylation. As a first step in such a direction, the reaction between human MICAL-MO and NADPH was studied in a stopped-flow under anaerobic conditions leading to the observation that the bound FAD is converted to the 2-electron reduced hydroquinone form without the detectable formation of intermediates. The apparent *K*_d_ for NADPH and the *k*_red_ were similar to those measured under steady-state conditions in the same buffer, including 10% glycerol (Table 2) that lowers *k*_cat_ and increases *K*_M_ for NADPH (Table 2). Thus, it appears that the enzyme NADPH oxidase activity is limited by the rate of the enzyme reductive half reaction.

Human MICAL-MO was found to be very sensitive to the ionic strength of the solvent and to the type of anions present, which all specifically lowered *k*_cat_/*K*_NADPH_.

Phosphate, chloride and acetate salts competed directly for NADPH binding regardless of the counter ion and with decreasing potency in the stated order. The lack of “special” effect of cations such as Mg^2+^ or Ca^2+^ is informative in the light of the role played by these ions *in vivo*. Interestingly, by varying the concentration of Tris and Bis-tris buffer, *k*_cat_/*K*_NADPH_ seemed to depend on electrostatics so that the dependence of the parameter from ionic strength could be well fitted with the Debye-Huckel equation [70]. The effects of anions on MICAL-MO further supports its functional similarity with enzymes of the PHBH family. In PHBH, anions compete with NADPH phosphate groups for the binding to positively charged residues on the protein (Figures 7 and 8). Furthermore, electrostatics plays an important role for both NADPH binding and catalysis [5,6,88].

**Figure 11:**
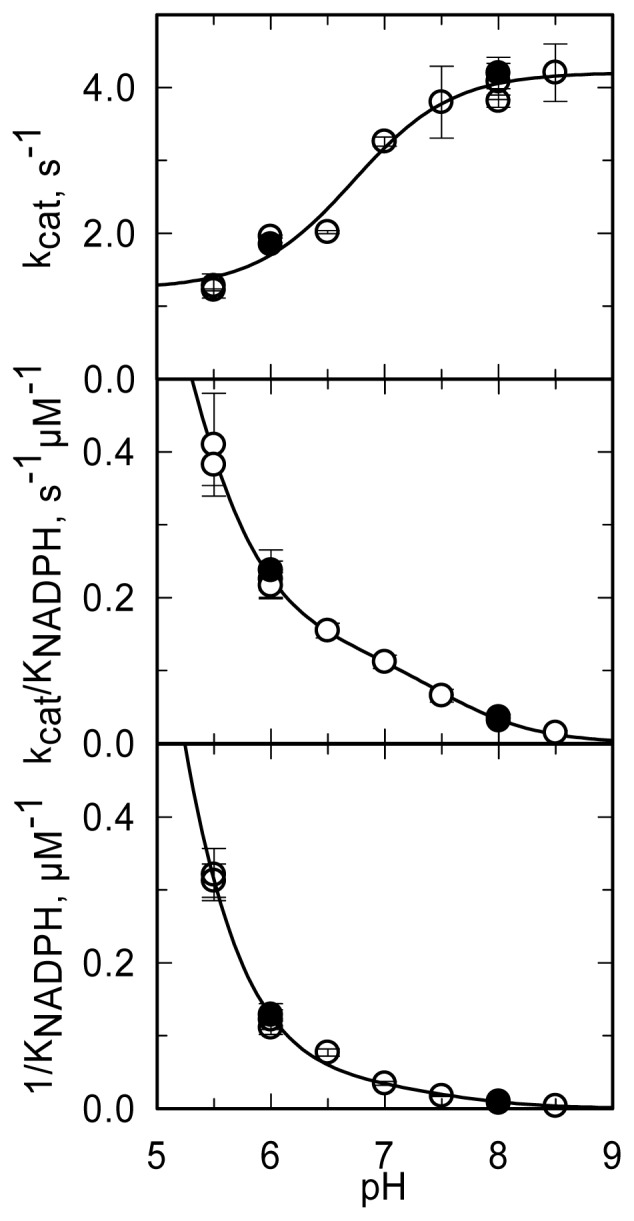
pH dependence of the kinetic parameters of the NADPH oxidase reaction of MICAL-MO. The steady-state kinetic parameters *k*_cat_, *K*_NADPH_ and *k*_cat_/*K*_NADPH_ were measured as described in Table 3, where the values of the calculated parameters are shown. Reprinted from [70] with permission from Elsevier.

The pH dependence of the steady-state kinetic parameters of the NADPH oxidase reaction was studied in a buffer system designed to maintain essentially constant ionic strength across the pH interval being explored and to minimize interference of specific anions (Figure 11, Table 3). *k*_cat_ increases from a low constant value (at low pH) to a limiting maximum value (at high pH) as a group with an apparent pK_a_ of 6.7 dissociates. *k*_cat_/*K*_NADPH_ and 1/*K*_NADPH_ showed similar pH dependencies. They decreased from high (non-limiting) values (at low pH) to low values (at high pH) as groups with apparent pK_a_ of 4.6–4.9 and ~7.5 deprotonated. The *k*_cat_, *k*_cat_/*K*_NADPH_ and 1/*K*_NADPH_ profiles (Figure 11) are all qualitatively similar to those obtained with PHBH when the interaction with NADPH was studied [88]. As proposed for PHBH, also in MICAL they may reflect an increased positive charge of the protein at low pH, which favors the interaction with the negatively charged NADPH. With respect to the group that needs to be deprotonated in order to observe maximum *k*_cat_, the group in the mouse MICAL-MO active site which is likely to exhibit a pK_a_ of 6.7 is His126 (Figure 9). This residue is conserved in all MICALs and may stabilize the “flavin in” conformation through H-bonds between its main chain atoms and the isoalloxazine N(3) and O(4) atoms. Neutralization of the side-chain of this residue upon deprotonation may favor the “flavin out” conformation, which is the conformation that allows hydride transfer from NADPH to the flavin N5 position.

Interestingly, *k*_cat_ and, to a greater extent, *k*_cat_/*K*_NADPH_ were found to be sensitive to solvent viscosity, consistently with the hypothesis that one or more of the conformational changes predicted to take place during MICAL-MO catalytic cycle is sufficiently slow to contribute to the determination of *k*_cat_ and *k*_cat_/*K*_NADPH_. With respect to the enzyme reductive half reaction, a minimal scheme including essential conformational changes would involve: (i) binding of NADPH to the oxidized species in the “flavin out” conformation with Trp400 stacking against the isoalloxazine ring; (ii) repositioning of Trp400 and of NADPH to reach the correct geometry for hydride transfer from the NADPH nicotinamide ring to the FAD isoalloxazine ring; (iii) hydride transfer with formation of FAD hydroquinone; (iv) switch from the “flavin out” to the “flavin in” conformation, which leads to formation of the tunnel connecting the surface of the protein with the active site (Figure 8); (v) completion of the monooxygenase/hydroxylation reaction.

This minimal scheme is certainly not sufficient to describe the details of the reaction since it does not take into account several relevant aspects such as, for example: (i) the existence of a third “open” conformation, as in PHBH; (ii) if NADPH binds to both “flavin out” and “flavin in” conformations, or just to the “out” state; (iii) whether NADP^+^ dissociates prior to the switch form the “out” to the “in” conformation, as in PHBH; (iv) whether the physiological substrate undergoing hydroxylation binds to both conformations and/or (v) how its binding affects the out/in states and NADPH/NADP^+^ binding and reactivity. Whether the NADPH oxidase activity of MICAL-MO requires the switch from the “out” to the “in” conformation is also not clear. In principle, reduced FAD may react with molecular oxygen when in the “out” conformation leading to production of hydrogen peroxide. In this respect a great deal of work has been done recently to elucidate how flavoenzymes react with oxygen (see [74] for a recent review and a collection of relevant references). Thus, it would be of great interest to apply similar experimental and theoretical approaches to the understanding of the reactivity with oxygen of MICAL in its various conformations.

Among essential steps towards the understanding of MICAL-MO physiological role is the identification of the physiological (hydroxylatable) substrate and of specific inhibitors.

A survey of potential substrates among amino acids, (poly)amines and aromatic compounds was done with the human MICAL-MO [70]. The molecules either had no effect on the reaction velocity or acted as inhibitors. Benzoate derivatives were mild inhibitors (estimated *K*_i_ values in the 0.1–1 mM range). They yielded competitive or non-competitive inhibition patterns with respect to NADPH, and their potency increased as the number of hydroxyl groups increased. This result correlated with the proposal that EGCG is a potent and selective inhibitor of mouse MICAL-MO ([4], *K*_i_, 2 μM). Testing the effect of this compound on human MICAL-MO confirmed that EGCG is a non-competitive inhibitor of MICAL-MO with similar effects on slopes and intercepts of double reciprocal plots (Table 4). However, EGCG was a less potent inhibitor of human MICAL-MO (*K*_i_, 17 μM) than reported for the mouse protein (*K*_i_, 2 μM, [4]). The difference may be related to the assay format (see above) by taking into account that EGCG, as a catechol, is a potent H_2_O_2_ scavenger. Interestingly, during spectrophotometric titrations, EGCG caused human MICAL-MO aggregation/denaturation at concentrations just above its K_i_ (17 μM, [70]), while no absorption changes were observed at lower concentrations. This result casts some doubt on the conclusion that EGCG is a selective inhibitor of MICAL-MO. It might even be suggested that some of the observed inhibitory effects of EGCG on semaphorin-induced growth cone collapse [2] may be due to its effect on protein aggregation/denaturation. The similarity of EGCG with xanthofulvin (SM-216289), which was reported to be a selective inhibitor of semaphorin of therapeutic interest [82,83], may lead to the proposal that also this compound may in part function as a radical scavenger or by denaturing semaphorin.

### 2.7. The Actin Depolymerizing Activity of MICAL

A great deal of work has been done to test the hypothesis that actin is a MICAL substrate. Fundamental *in vitro* experiments were done using Drosophila MICAL forms comprising the MO (MICAL-MO) and both the MO and CH domains (MICAL-MOCH). The *in vitro* work complemented genetic studies in Drosophila in which the actin-rich bristles were used as a model to study the effect of MICAL forms on actin organization [52,53].

**Figure 12:**
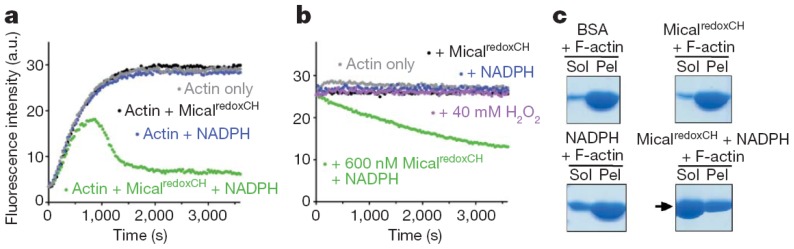
Effect of Drosophila MICAL forms and NADPH on the actin polymerization state. (**a**) Pyrene-labelled G-actin (1.1 μM final concentration) was incubated with Drosophila MICAL-MOCH (600 nM) and/or NADPH (100 μM) in G buffer (5 mM Tris-HCl pH 8.0, 0.2 mM CaCl_2_, 0.2 mM ATP and 1 mM DTT). Polymerization of actin was induced by adding 5 mM Tris-HCl pH 7.5, 50 mM KCl, 2 mM MgCl_2_, 1 mM EGTA, 0.5 mM DTT, and 0.2 mM ATP. Fluorescence intensity was immediately monitored at 407 nm with excitation at 365 nm by a fluorescence spectrophotometer (Spectra max M2; Molecular Devices); (**b**) Drosophila MICAL-MOCH (Mical redoxCH in the figure), NADPH, and/or hydrogen peroxide were added to pre-polymerized pyrene-labelled F-actin (1.1 μM) and fluorescence intensity was immediately monitored as described above; (**c**) Pre-polymerized F-actin (18.6 μM) was incubated with Drosophila MICAL-MOCH (2.4 μM final concentration), and/or NADPH (200 μM final concentration) for 30 min at room temperature. Then, the samples were subjected to high-speed centrifugation at 150,000 × *g* for 1.5 h at 24 °C. Supernatants and resuspended pellets were analysed by SDS-PAGE followed by Coomassie blue staining. Experimental conditions have been derived from the procedures described in the Supplementary information of [53]. Reprinted by permission from Macmillan Publishers Ltd.: [Nature] ([53]), copyright (2010).

Several tests were used to study the effect of Drosophila MICAL forms on globular (monomeric) actin (G-actin) and on the filamentous form (F-actin). Similar results were obtained with the Drosophila MICAL-MO and MICAL-MOCH forms indicating that the CH domain is not essential for MICAL-actin interaction. Both MICAL forms were found to co-sediment with F-actin, demonstrating an interaction between the proteins (Figure 12). When G-actin was allowed to polymerize in the presence of MICAL forms and NADPH, a significant fraction of actin (and MICAL) was found in the supernatant from a high-speed centrifugation indicating that MICAL caused F-actin depolymerization. By exploiting the fact that pyrenyl-actin fluorescence is significantly greater in F-actin than in G-actin (λ_ex_, 365 nm; λ_em_, 407 nm), it was shown that Drosophila MICAL-MO and MICAL-MOCH interfere with actin polymerization and cause actin depolymerization in a fashion that is dependent on NADPH and MICAL concentration (Figure 12). In these experiments, initially, fluorescence increases due to the prevailing actin polymerization. Eventually, fluorescence decreases due to actin depolymerization until a steady-state is reached. Addition of MICAL forms and NADPH to pre-polymerized pyrene-labeled F-actin led to observing fluorescence decrease, consistently with actin depolymerization. In these and several other experiments presented by Hung *et al*. [52,53], the observed depolymerizing activity was associated with the MO domain, as no differences were observed when experiments were carried out with the MICAL-MO or MICAL-MOCH forms. Importantly, MICAL appears to interact along the F-actin filament rather than at one of its ends and several actin bundling and capping proteins have no effect on MICAL depolymerizing action [52,53]. Interestingly, it was also shown that direct contact between MICAL forms and F-actin was required for the depolymerization [52]. This finding led the authors to rule out that MICAL’s effect on F-actin is due to diffusible hydrogen peroxide resulting from the MICAL’s NADPH oxidase activity, thus supporting the hypothesis that MICAL catalyzes a monooxygenase reaction. However, it cannot be ruled out that MICAL may catalyze a NADPH oxidase reaction when bound to F-actin and that it is the high local hydrogen peroxide concentration that causes F-actin depolymerization. Finally, by reisolating actin after incubation with MICAL-MOCH and NADPH under polymerization conditions, it was shown that actin was unable to repolymerize [52]. Modification of Met44 and Met47 into methionine sulfoxide was detected by mass spectrometry in MICAL-treated actin. Met44 was identified as the site of specific modification by generating Drosophila Met44Leu and Met47Leu actin variants. The proteins were able to polymerize and to bind MICAL in the absence of NADPH, but the Met44Leu and the Met44Leu/Met47Leu variants were no longer sensitive to MICAL and NADPH [52].

Met44 is in the actin d-loop at the pointed end of the actin monomer. In G-actin, the loop is very flexible and Met44 is among the residues that are the most sensitive to oxidation [89–92]. High resolution models of F-actin have not been obtained yet making it difficult to discuss Met44 role in F-actin and its accessibility to MICAL. In the F-actin model of Oda *et al*. [93], the d-loop makes extensive (both electrostatic and hydrophobic) longitudinal contacts with the adjacent monomer along the filament, which is a left handed, two-chained long helix [92]. Thus, the d-loop and its precise conformation are believed to be important for F-actin stability so that chemical modifications of one or more of its residues may indeed destabilize the filament. Although Met44 is partially protected from oxidizing agents in F-actin [90], it may lie toward the outside of the F-actin structure (with Met47, [90]) so that it may be accessible to MICAL-MO. This may also be possible in the light of the conformational flexibility of the d-loop in actin filaments as tested by cross-linking experiments with forms of yeast actin in which the d-loop was subjected to Cys-scanning [94]. In this respect it is of interest that expression of the Met44Cys yeast actin variant in yeast led to no viable colonies supporting a crucial role of this residue [94]. For MICAL-MO to modify actin Met44, MICAL should be able to extract the residue from the interface between actin monomers in the filament. Conversion of Met44 into methionine sulfoxide would locally destabilize the filament promoting its fragmentation. Modeling studies on actin filaments indicated a conformational heterogeneity of actin monomers along the filament, which may affect its local stability [95]. Thus, Met44 could be accessible to MICAL at least in some parts of the polymer. In this respect, similar modeling studies may greatly contribute to the understanding of MICAL-actin interactions.

In support of the identification of Met44 as the actin residue specifically targeted by MICAL is the fact that introducing the Met44Leu variant into *Drosophila* bristles, a phenotype similar to that obtained with a MICAL loss-of-function mutant is observed. Interestingly, the dominant Met44Thr mutation of skeletal muscle actin is associated with nemaline myotrophy, in which actin accumulation and aggregation are observed [96].

**Figure 13:**
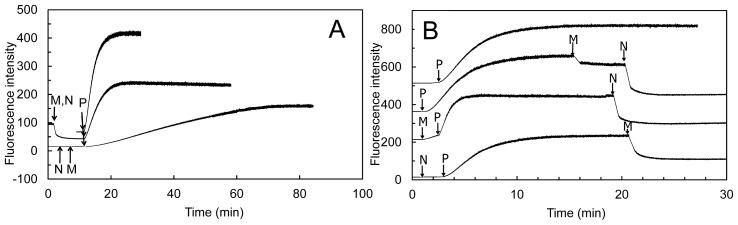
Effect of human MICAL-MO on actin polymerization state as determined fluorimetrically. (**A**) The polymerization state of pyrene-labelled G-actin (4.1 μM) in the presence or absence of NADPH (100 μM, N) and human MICAL-MO (660 nM, M) was monitored fluorimetrically in a Cary Eclipse (Varian) fluorimeter. The polymerization was induced by addition of 1/10 volume of polymerization buffer (P, 50 mM Tris/HCl, pH 7.5, 500 mM KCl, 20 mM MgCl_2_, 5 mM DTT, 10 mM ATP) at the indicated time to the solution in G-buffer (5 mM Tris/HCl, pH 8.0, 0.2 mM CaCl_2_, 0.2 mM ATP, 1 mM DTT); (**B**) Fluorescence-monitored polymerization and depolymerization of actin (2.3 μM) in the presence of NADPH (100 μM, N) and human MICAL-MO (660 nM, M) added at the indicated times. In the panels the traces have been offset for graphical purposes. Reprinted from [70] with permission from Elsevier.

**Figure 14:**
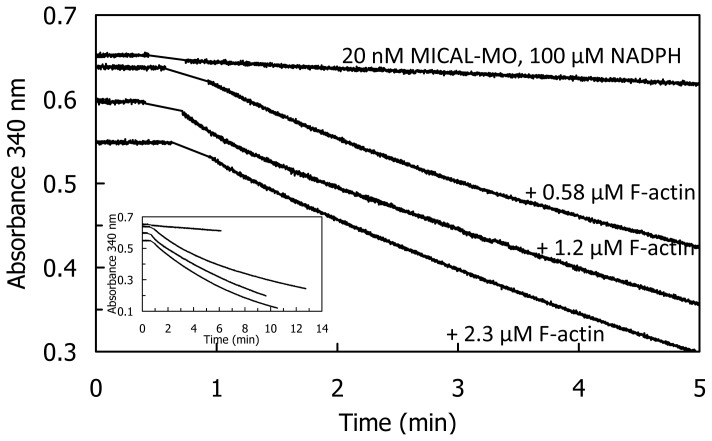
Time-course and extent of NADPH oxidation in the presence of human MICAL-MO and varying F-actin concentrations. Human MICAL-MO (20 nM) and NADPH (100 μM) were added to a solution containing the indicated concentrations of F-actin in F-buffer (9 mM Tris/HCl, pH 8.0, 0.18 mM CaCl_2_, 1.1 mM ATP, 1.3 mM DTT, 45 mM KCl, 1.8 mM MgCl_2_). The absorbance changes at 340 nm were monitored. The inset shows the same traces but on a different time-scale. Reprinted from [70] with permission from Elsevier.

**Figure 15:**
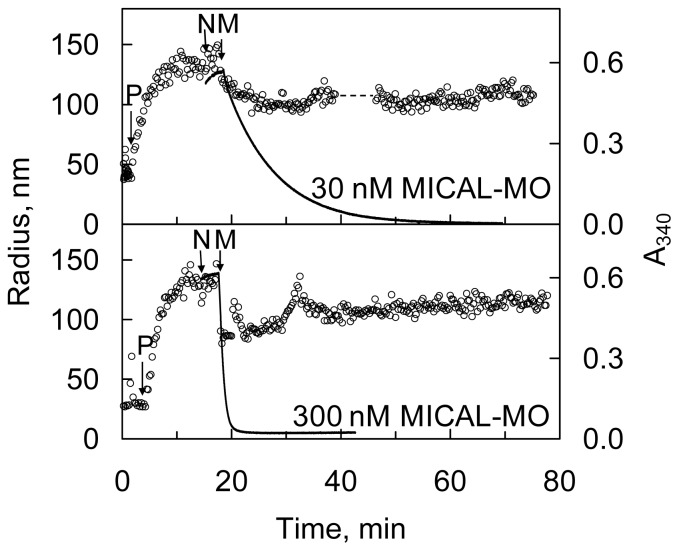
Monitoring the changes of the aggregation state of actin in solution in the presence of human MICAL-MO and NADPH by dynamic light scattering. Polymerization of G-actin (5 μM) was induced by adding 1/10 volume of polymerization buffer (P) at 25 °C. The mean radius of the particles in solution was determined with a Dynapro MS/X instrument (Protein Solutions, Lakewook, NJ, USA) by acquiring signals every 15 s (○). NADPH (100 μM, N) and MICAL-MO (M, 30 or 300 nM) were added at the indicated times and the reaction was monitored by dynamic light scattering (DLS). Solid lines: oxidation of NADPH (100 μM) monitored at 340 nm under identical conditions. The *A*_340_ traces are offset to match the time of enzyme addition to the solution containing F-actin and NADPH in the DLS experiment. Reprinted from [70] with permission from Elsevier.

The human MICAL-MO was also found to exhibit an F-actin depolymerizing activity [70]. The reaction was monitored fluorimetrically by exploiting the different fluorescence intensity of pyrene-labeled G- and F-actin (Figure 13) and by sedimentation assays. Dynamic light scattering (DLS) was also used to determine the average radius of the species in solution. The time-course and extent of NAD(P)H oxidation were monitored spectrophotometrically (Figures 14 and 15).

Addition of human MICAL-MO to pre-polymerized pyrenyl-labeled actin, in a buffer stabilizing F-actin (F-buffer), after NADPH, led to a decrease of fluorescence, which was dependent on MICAL and NADPH concentration (Figure 13). Also some human MICAL-MO was found to co-sediment with F-actin. When pre-polymerized F-actin was incubated with human MICAL-MO and NADPH in F-buffer, a fraction of actin was found in the supernatant after high-speed centrifugation confirming MICAL- and NADPH-dependent F-actin depolymerization.

The rate and extent of NAD(P)H consumption were monitored spectrophotometrically in the presence of G- and F-actin demonstrating that F-actin, but not G-actin, significantly stimulates NAD(P)H oxidation by lowering the *K*_M_ value for NADPH (50-fold) and increasing k_cat_ (3- or 6-fold at 2.4 μM actin or extrapolated at infinite actin concentration, respectively) when compared to the values measured in the absence of actin, but under the same buffer conditions (Table 5) resulting in a ~500–800– fold *k*_cat_/*K*_NADPH_ increase.

These results are those expected for an authentic monooxygenase in the presence of its substrate. A *K*_M_ for F-actin of 4.7 μM was determined at 300 μM NADPH. A decrease of the intensity of the signal was also detected by DLS. It paralleled the decrease of absorbance at 340 nm confirming the F-actin depolymerizing effect of MICAL-MO in the presence of NAD(P)H (Figure 15). Some technically critical points were highlighted by comparing the experiments done with the human [70] and the Drosophila proteins [52,53]. First, and as expected, it was found that NAD(P)H interfered with fluorescence monitoring of pyrene-labeled actin polymerization state (Figure 13). At the wavelength used to excite actin-bound pyrene (λ_ex_, 365 nm), NAD(P)H absorbs a significant fraction of light causing a drastic decrease of the intensity of the light emitted by pyrene at 407 nm in F-actin. Thus, the fluorescence intensity values alone may not fully reflect the rate and extent of pyrenyl-actin polymerization/depolymerization. Secondly, human MICAL-MO appeared to be more active than the Drosophila forms (compare Figure 12 and Figures 13, 14, 15). Almost stoichiometric amounts of Drosophila MICAL-MO or MICAL-MOCH were routinely used to carry out the depolymerization reaction, which was completed in ~60 min, namely 600 nM MICAL form and 1–5 μM actin monomer. With a similar concentration of human MICAL-MO, the reaction was completed within 2–5 min from MICAL addition even in the absence of actin (*i.e*., in the NADPH oxidase activity). As a consequence of this observation, all experiments with the human protein were carried out with 10–100 nM MICAL-MO (*i.e*., with catalytic amounts of MICAL) in the presence of pre-polymerized 2–5 μM actin and in buffer stabilizing the filamentous actin form. Furthermore, in several of the experiments with the Drosophila protein, it appears that G-actin was mixed with MICAL forms and NADPH prior to inducing polymerization. With this assay format, it may be difficult to know how much residual NADPH is present in solution by the time actin polymerization is initiated.

More importantly, inspection of the time-course and extent of NADPH consumption in the presence of human MICAL-MO (Figures 14 and 15, [70]) revealed an as yet unsolved puzzling aspect. When experiments like those shown in Figure 14 and 15 were carried out, the extent and time-course of NADPH oxidation was different from those expected. Assuming that MICAL-MO catalyzes the hydroxylation of one specific actin residue, with a 1:1 stoichiometry of NADPH being oxidized per actin monomer being modified, one would have predicted biphasic traces. Initially, MICAL would behave as a monooxygenase. The rate of NADPH consumption would be fast and the extent of the phase should be lower or equal to the total actin monomer concentration. A second slower phase of NADPH oxidation should follow due to the MICAL NADPH oxidase reaction with residual oxygen.

On the contrary, although traces showed some biphasicity, which could not be clearly correlated with the total actin concentration present, the second phase was much faster than that measured in the NADPH oxidase activity under the same conditions (Figure 14) and led to oxidation of all NADPH present. The observed extent and time-course of NADPH consumption are consistent with a case of substrate recycling suggesting that the effect of MICAL (and NADPH) on F-actin may be reversible. In support of this hypothesis is the fact that when the reaction was monitored by DLS, actin was never fully converted to the monomeric form, as judged by the average size of the particles in solution (Figure 15). An allusion to this possibility is made also by Hung *et al*. [52], perhaps taking into account the results obtained with the human MICAL-MO [70].

The same extent and time-course of NADPH oxidation were obtained when assays were done in buffers that do not stabilize F-actin, and in the absence of dithiothreitol (DTT). The latter, which is included in the buffers to protect actin from non specific oxidation, efficiently scavenges any hydrogen peroxide present, but may also reverse oxidation of Cys residues. How one could reconcile the extent and rate of NADPH consumption observed with the human MICAL-MO [69] and the irreversible depolymerization of actin due to quantitative chemical modification of actin monomers reported by Hung *et al*. [52] with the Drosophila protein is not clear. Thorough studies of the reaction are indeed required. However, it is unlikely that the effect on actin is due to hydrogen peroxide generation on the basis of independent observations. Only high H_2_O_2_ concentrations lead to actin depolymerization as judged from decrease of fluorescence of pyrenyl-labeled actin [52,53] and DLS (unpublished data). Direct contact between Drosophila MICAL forms and F-actin was required to cause depolymerization [52]. Control experiments also ruled out that NADP^+^ alone or in combination with H_2_O_2_ may be responsible for actin depolymerization [53]. However, as pointed out before, a high local hydrogen peroxide concentration produced by MICAL oxidase activity when bound to F-actin, may be sufficient to chemically modify one or more actin residues and cause filaments’ depolymerization.

## 3. Perspectives

MICAL appears to directly link semaphorin signaling to a chemical modification of actin subunits when assembled in filaments, which causes actin depolymerization. This very valuable information is by no means sufficient to clarify the complex series of events that follow the semaphorin/plexin interaction nor how cytoskeleton dynamics is controlled (see, e.g., [8,9,16,28,31] and references therein). However, clarifying how MICAL works through further *in vitro* studies may eventually contribute to the understanding of these important pathways.

The structural similarity between MICAL *N*-terminal region and FAD containing monooxygenases of the PHBH family [3,4] as well as the spectroscopic and kinetic studies done with the human MICAL1 *N*-terminal flavoprotein domain strongly support that the protein is a monooxygenase [70], which catalyzes a NAD(P)H-dependent hydroxylation reaction. The work of Hung *et al*. [52,53] indicates that actin Met44 is the physiological substrate. However, the details of MICAL’s catalytic activity still need to be clarified. A few selected issues will be discussed below.

The actin depolymerizing effect of MICAL-MO has been confirmed with the human protein, but the origin of the unexpected time-course and extent of NADPH consumption observed with human MICAL-MO needs to be elucidated. Drosophila MICAL has been shown to bind along the actin filament and produce short F-actin filaments [52] upon chemical modification of Met44. Thus, assuming that such short actin filaments are also MICAL’s substrates, one would expect an increase of the rate of NADPH oxidation as more F-actin fragments are generated in solution resulting in an increase of MICAL effective substrate concentration. Still a 1:1 stoichiometry of NADPH being oxidized and actin monomers should be observed during the faster reaction phases. The hypothesis that the observed extent and time-course of NADPH consumption is due to the sum of the actin hydroxylating/depolymerizing activity and of an enhanced NADPH oxidase activity in the presence of actin fragments needs to be tested by, e.g., correlating NADPH oxidation, oxygen consumption and hydrogen peroxide production during the reaction. These and several other types of experiments will be enormously complicated by the protein nature of the substrate and its different aggregation states in solution. However, G-actin does not stimulate NADPH consumption and no H_2_O_2_ production was detected at the end of a reaction in which NADPH (100 μM) was incubated with F-actin (4 μM) in the absence of DTT and NADPH oxidation was allowed to reach completion (unpublished data). That loss of absorbance at 340 nm is due to NADPH oxidation rather than some other reaction was also recently established by monitoring NADPH regeneration upon addition of glucose 6-phosphate and glucose 6-phosphate dehydrogenase at the end of the reaction of MICAL, NADPH and F-actin (unpublished data).

The irreversible F-actin depolymerization following chemical modification of a protein side-chain observed with the Drosophila protein, and the substrate recycling behavior observed with the human MICAL-MO might be reconciled assuming a dual catalytic activity of MICAL flavoprotein domain. MICAL-MO could cause actin filaments fragmentation and/or release of actin from the filament through its monooxygenase reaction. However, it could also reverse the chemical modification in a NADPH-dependent reaction. Verifying this hypothesis will require, first, to confirm the selective modification of actin Met44 by MICAL’s from sources other than Drosophila. Then, methods to properly handle and analyze the protein reaction product while avoiding artifacts will need to be developed. The substrate recycling behavior observed with the human protein could also be explained by assuming that MICAL-MO depolymerizes F-actin by binding to it in one of its conformations (e.g., the “flavin out” conformation, typical of the oxidized form) and destabilizing the filament by undergoing a conformational change associated with the switch to its alternative (“flavin in”) conformation upon reduction. However, this hypothesis would be in contrast with the irreversibility of actin depolymerization and the finding of the chemically modified Met44 observed with the Drosophila protein [52].

It also needs to be established if MICAL1-3 exhibit the same catalytic activity towards the same (protein) substrate (and they just differ with respect to activatory/inhibitory mechanisms), if they differ for the substrate specificity (and type of targeted residue) and/or if they differentially control their oxidase and monooxygenase activities.

A series of experiments suggested that CRMP proteins are interactors of MICAL1 and that they may also be MICAL substrates [24]. In co-immunoprecipitation experiments CRMP1 was found to interact with MICAL1 in the region comprising the flavin, CH and LIM domains. The extent of Cos7 cell contraction, which provides a useful method to study semaphorin signaling in non-neuronal cells, was used to monitor the CRMP-MICAL interaction. Overexpression of MICAL1 forms lacking the *C*-terminal 290–600 residues in such background led to cell contraction similarly to that obtained by expressing a constitutively active plexin A mutant. Thus, it was concluded that the *C*-terminal 290 residues (including the Pro-rich region) autoinhibits MICAL1 activity so that the isolated MO domain is constitutively active. Accordingly, coexpressing the two 1–760 and 761–1048 protein fragments restored the phenotype observed with the full-length protein. It was also shown that the MO domain was necessary to determine cell contraction and to ensure a response to semaphorin further supporting a link between its catalytic activity and semaphorin signaling. The cell collapse was enhanced by co-expressing CRMP1 and MICAL1 forms supporting an interaction between the proteins in the cells. The amount of hydrogen peroxide produced in cells expressing MICAL1 forms was also quantified in crude extracts and in the presence of NADPH. A high level of hydrogen peroxide production by a MICAL form comprising residues 1–760, *i.e*., the MO, CH and LIM domains (Figure 1) was observed. Hydrogen peroxide production was quenched in the presence of the *C*-terminal region of MICAL and by CRMP1. The effect of CRMP on hydrogen peroxide production led to the proposal that it may be a substrate of MICAL’s monooxygenase activity (Figure 3). As an alternative, CRMP could inhibit MICAL-MO’s NADPH oxidase activity by preventing NADPH or oxygen binding to the catalytic site. Finally, CRMP may present to MICAL’s catalytic site the substrate to be hydroxylated. This molecule could be one of CRMP protein ligands or a small molecule. CRMP is structurally related to dihydropirimidinases, although it is devoid of the corresponding catalytic activity [16,24,97,98]. The small molecule could be harbored in the site corresponding to the dihydropyrimidinase active center.

The fact that some CRMP forms can be produced in large quantities [97,99] will allow to test both the hypothesis that CRMP is a MICAL’s substrate undergoing hydroxylation (or oxidation as proposed by [54]) and/or that it substitutes for the MICAL’s *C*-terminal domain preventing the (harmful) NADPH oxidase activity of the MO domain. The inhibition of the oxidase activity would be released when MICAL’s C-terminal domain is engaged in the interaction with the cytoplasmic region of plexin [16,24]. The work of Schmidt *et al*. [24], which was later confirmed by Giridharan *et al*. [25], indicates a role of MICAL’s additional domains in controlling the NADPH oxidase activity of the *N*-terminal catalytic region. A true monooxygenase should be virtually inactive in the absence of the substrate to be hydroxylated. Thus, the observed NADPH oxidase activity of the isolated MO domain could arise from expression of just one part of the protein. Also this hypothesis could be tested after production of MICAL forms comprising the various domains. That the production of the MICAL-MOCH form is feasible has been demonstrated for the Drosophila [52,53] and human proteins (Vitali, T. and Vanoni, M.A., unpublished data).

Understanding the conformational changes brought about by the presence of the various MICAL’s domains and their interactors, as well as those that are predicted to take place within the MO domain during the catalytic cycle is one of the major experimental challenges of future studies on MICAL. In this respect, combining experimental and computational work may be of great help as already demonstrated in the actin [95,100–103] and flavoprotein fields [77,78,104–107]. The fact that MICAL-MO exhibits a detectable NADPH oxidase activity may also allow us to gain information on the enzyme reductive half reaction of monooxygenases of the PHBH class without the complication of the presence of the hydroxylatable substrate. The pH dependence and the sensitivity of the reaction to anions and to solvent viscosity may allow us to trap intermediates along the reaction path.

A better understanding of the catalytic properties of MICAL and of their modulation will contribute to shed light on semaphorin signaling and cytoskeleton dynamics in health and disease. Furthermore, it may be possible to obtain information useful to interfere with these processes with therapeutic purposes.

## Figures and Tables

**Table 1:** Summary of MICAL sequences as retrieved from databanks. The identification was done by taking into account annotations and direct sequence comparisons. When several isoforms for each one of the predicted proteins exist on the basis of alternative splicing and comparison of cDNA and DNA clones deposited in databanks, the putative full-length protein was selected following sequence comparisons within and among the MICAL1–3 classes using BLAST and ClustalW2 or Clustal Omega [61]. The accession codes of isoforms are given in a few instances.

Protein	Organism	Chromosome location	Nucleotide	Protein	Length (residues)	Gene ID	UniPro/SwissProt	Remarks
MICAL1	human	6q21	NM_022765	NP_073602	1067	64780	Q8TDZ2	Figure 4
MICAL2	human	11p15	NM_014632	NP_055447	1124	9645	O94851	Figure 4
MICAL3	human	22q11.21	NM_015241	NP_056056	2002	57553	Q7RTP6	Figure 4
MICAL1	mouse	10 B1-B2	NM_138315	NP_612188	1048	171580	Q8VDP3	Figure 4
MICAL2	mouse	7E3	NM_001193305	NP_001180234	1102	320878	8QBML1	isoform A; Figure 4
MICAL3	mouse	6F1	NM_001270475	NP_001257404	1993	194401	Q8CJ19	isoform 1; Figure 4
MICAL1	rat	20q12	NM_001106397	NP_001099867	1047	294520	D3ZBP4	Figure 4
MICAL2	rat	1q33		EDM_17817	1103	365352	D4A1F2-2	Figure 4
MICAL3	rat	4q42	NM_001191085	NP_001178014	1997	362427	-	Figure 4
MICAL1	guinea pig	-	XM_003466042	XP_003466090	1058	100715118	H0V032	Figure4
MICAL2	guinea pig	-	XM_003465749	XP_003465749	1105	100735168	-	Figure 4
MICAL3	guinea pig	-	XM_003461762	XP_003461810	2005	100726239		Figure 4
MICAL1	macaque	4	XM_002803949	XP_002803995	1065	698737		Figure 4
MICAL2	macaque	14		EEH23095	1124	701439		Figure 4
MICAL3	macaque	10	XM_001103660	XP_001103660	2001	710292		Figure 4
MICAL1	D. rerio	23	XM_003201226	XP_003201274	1214	568573	E7F9T0	Predicted as MICAL3, Figure 4
MICAL2	D. rerio	25	JX291155	AFS28884	1120	569564		Figure 4
MICAL3	D. rerio	18			1994	567456	F1QH17.2	Authentic according to UniPro; Figure 4
MICAL	drosophila	3R	AF520715	AAM55244	4723	41225	Q86BA1	long isoform differing from others for interdomain regions; Figure 4

**Table 2:** Summary of the kinetic properties of the NADPH oxidase reaction of human MICAL-MO. Assays were carried out at 25 °C in the presence of the indicated NAD(P)H concentration ranges and additions in 20 mM Hepes/KOH buffer, pH 7.0. The last line reports the apparent *K*_d_ for NADPH [86] and *k*_red_ calculated from an anaerobic stopped-flow experiment in which MICAL-MO was reacted with varying concentrations of NADPH. Data are from [70].

NADPH, μM	NADH, μM	NaCl, M	Glycerol, %	*K*_M_, μM	*k*_cat_, s^−1^	*k*_cat_/*K*_M_, s^−1^ mM^−1^
4–160				28 ± 2	4.0 ± 0.1	143 ± 11
40–650		0.1		499 ± 28	2.6 ± 0.1	5.2 ± 0.4
20–200				28 ± 3	4.4 ± 0.1	157 ± 17
	80–670			580 ± 24	0.28 ± 0.01	0.48 ± 0.03
10–300				26 ± 4	3.9 ± 0.1	150 ± 23
10–300			10	93 ± 11	2.9 ± 0.1	31.2 ± 4
	(reductive half reaction)		10	56 ± 7.3	3 ± 0.1	

**Table 3:** pH dependence of the steady-state kinetic parameters of the NADPH oxidase activity of human MICAL-MO. The pH dependence of the kinetic parameters of the NADPH oxidase reaction of MICAL-MO was measured at 25 °C in the presence of a mixed buffer composed of 10 mM acetic acid, 5 mM imidazole and 5 mM Tris. pH was adjusted with NaOH and ionic strength was kept constant at 10 mM with the addition of suitable amounts of sodium acetate. The *k*_cat_ values increase from a lower limit (Lim1) at acidic pH to an upper limit (Lim2) as a group with a pK_a_ of ≈6.7 dissociate. *k*_cat_/*K*_NADPH_ and 1/*K*_NADPH_ decreased from a (poorly defined) upper limit (Lim1) at low pH to a lower limit (Lim2) at high pH as groups with pK_a_ of 4.6–4.9 and ≈7.5 dissociate.

Parameter	Lim 1	Lim2	pK_a1_	pK_a2_
*k*_cat_, s^−1^	1.2 ± 0.15	4.2 ± 0.1	6.73 ± 0.13	
*k*_cat_/*K*_NADPH_, s^−1^μM^−1^	1.3 ± 0.87	0.13 ± 0.02	4.93 ± 0.13	7.50 ± 0.13
1/*K*_NADPH_, μM^−1^	2.6 ± 0.36	0.031 ± 0.007	4.59 ± 0.01	7.54 ± 0.35

**Table 4:** Inhibition of the human MICAL-MO NADPH oxidase activity by NADP^+^ and EGCG. Assays were carried out at 25 °C in the presence of the indicated NAD(P)H concentration ranges and additions in 20 mM Hepes/KOH buffer, pH 7.0. Other conditions are as in Table 2. Data are from [70].

NADPH, μM	Inhibitor, μM	NaCl, M	K_M_, μM	k_cat_, s^−1^	K_i_, μM
15–160	NADP^+^, 0–0.15	0	24.3 ± 2.1	3.6 ± 0.1	77 ± 8
0–100	EGCG, 0–50	-	25 ± 2	3.9 ± 0.1	17 ± 0.6

**Table 5:** Effect of G- and F-actin on the steady-state kinetic parameters of the reaction of human MICAL-MO. The steady-state kinetic parameters of the reaction catalyzed by human MICAL-MO in the absence and presence of G- and F-actin were measured at 25 °C in 20 mM Hepes/KOH buffer, pH 7.0 (Hepes), G-buffer (5 mM Tris/HCl, pH 8.0, 0.2 mM CaCl_2_, 0.2 mM ATP, 1 mM DTT) or and F-buffer (9 mM Tris/HCl, pH 8.0, 0.18 mM CaCl_2_, 1.1 mM ATP, 1.3 mM DTT, 45 mM KCl, 1.8 mM MgCl_2_). The substrates concentrations or concentration ranges are indicated. The *K*_M_ is that calculated for the varied substrate. Data are from [70].

NADPH, μM	MICAL, nM	Buffer	Actin, μM	*K*_M_, μM	*k*_cat_, s^−1^	*k*_cat_/*K*_m_, s^−1^ μM^−1^
10–300	30	Hepes	-	19 ± 2	3.1 ± 0.1	0.163 ± 0.018
10–300	30	G-buffer	-	102 ± 8	3.4 ± 0.1	0.034 ± 0.003
10–300	75	G-buffer	G, 3.3	96 ± 28	4.5 ± 0.6	0.047 ± 0.015
10–300	120	F-buffer	-	555 ± 118	2.6 ± 0.4	0.005 ± 0.001
10–300	20	F-buffer	F, 2.4	11 ± 3	12.3 ± 0.5	1.12 ± 0.31
300	20	F-buffer	F, 7–85	4.7 ± 1	19.5 ± 1	4.2 ± 0.9
